# Acute Effects of Interrupting Prolonged Sitting on Glucose, Insulin and Lipid Metabolism in Healthy Populations: A Three-Level Meta-Analysis

**DOI:** 10.3390/metabo16080528

**Published:** 2026-07-27

**Authors:** Mingnan Zhuang, Yufei Li, Zhengqi Qiu, Xueqin Zhang

**Affiliations:** 1 Department of Applied Psychology, School of Health Management, Guangzhou Medical University, Guangzhou 510182, China; zmingnan027@stu.tjus.edu.cn; 2 School of Sports Training, Tianjin University of Sport, Tianjin 301617, China; 3 Shenzhen Institutes of Advanced Technology, Chinese Academy of Sciences, Shenzhen 518055, China; liyufei@greatteam.org.cn

**Keywords:** sedentary behavior, sitting interruption, postprandial glucose, insulin, triglycerides, meta-analysis, randomized crossover trials

## Abstract

**Background/Objectives:** Prolonged sedentary behavior is associated with adverse cardiometabolic risk, but the acute metabolic effects of interrupting sitting remain incompletely quantified in apparently healthy populations. The objective was to evaluate the acute effects of interrupting prolonged sitting on glucose, insulin and lipid metabolism and to identify potential protocol-level moderators. **Methods:** We conducted a three-level meta-analysis of randomized crossover trials comparing structured sitting interruptions with uninterrupted sitting in apparently healthy participants, an approach used to account for the statistical dependency among multiple effect sizes reported within individual studies. PubMed, Embase, Web of Science, Scopus and Cochrane Library were searched from inception through March 2026. Standardized mean differences (Hedges’ *g*) were calculated using random-effects models with robust variance estimation. Subgroup analyses and meta-regression examined interruption modality, intensity, frequency and duration. Certainty of evidence was assessed using GRADE. **Results:** The evidence base comprised 25 publications representing 23 independent cohorts; primary outcome analyses included 23 cohorts (135 effect sizes). Sitting interruption produced small but significant reductions in glucose (*g* = −0.28, 95% CI −0.49 to −0.08; PI −1.15 to 0.59) and insulin (*g* = −0.22, 95% CI −0.36 to −0.07; PI −0.51 to 0.07). No significant effect was observed for triglycerides (*g* = −0.04, 95% CI −0.14 to 0.07), whereas the exploratory estimates for non-esterified fatty acids (NEFA) and C-peptide were inconclusive. Subgroup analyses did not identify any statistically significant moderators of glucose, insulin or triglyceride responses; all subgroup estimates drawn from fewer than five independent cohorts are exploratory and hypothesis-generating. Meta-regression identified no statistically significant linear moderators. Certainty of evidence was low for all three primary outcomes (glucose, insulin and triglycerides) and very low for the exploratory outcomes (NEFA and C-peptide). **Conclusions:** Interrupting prolonged sitting may produce modest acute improvements in glucose and insulin responses in apparently healthy participants but does not reliably affect triglyceride metabolism. Given the low certainty of the current evidence, sitting interruption may be considered a low-burden adjunctive behavior for reducing uninterrupted sitting time. However, the current evidence primarily supports the hypothesis that interrupting prolonged sitting is a promising behavioral strategy rather than an evidence-based clinical recommendation, and its metabolic effects require confirmation in adequately powered, higher-quality trials.

## 1. Introduction

Sedentary behavior is a distinct physiological exposure, not simply the absence of moderate-to-vigorous exercise. Prolonged sitting suppresses muscle contractile activity, alters vascular and substrate-delivery conditions, and can acutely disturb glucose, insulin and lipid handling even when total daily energy expenditure changes little [1,2]. This distinction is especially relevant in apparently healthy adults. Postprandial metabolism can reveal early cardiometabolic strain before fasting glucose, body mass or overt disease changes. Repeated excursions in glucose, insulin and triglyceride-rich lipoproteins may therefore mark a metabolic cost of uninterrupted sitting that total activity volume alone can miss.

Recent evidence strengthens this prevention-oriented rationale. In free-living adults without diagnosed diabetes, postprandial glucose responses vary with daily movement, sleep and diet, and higher physical activity around meals is associated with lower glucose excursions [3]. Prospective cohorts further suggest that sedentary time is not a benign background exposure: greater weekly sitting time has been associated with incident diabetes in middle-aged and older Chinese adults, while accelerometer-measured sedentary behavior predicts future cardiovascular disease risk [4,5]. Systematic evidence on sedentary-pattern metrics also indicates that long uninterrupted sitting bouts are associated with non-communicable disease and mortality risk [6]. The public health implication is not simply that people should exercise more; rather, the temporal distribution of low-movement behavior may itself be metabolically relevant. Large population data showing potential benefit when sedentary leisure time is reallocated to other discretionary movement behaviors reinforce this behavioral substitution perspective [7].

Focusing on apparently healthy participants is therefore not a dilution of clinical relevance; it is a way to study the earliest reversible stage of metabolic strain. In this group, acute postprandial excursions can be interpreted without the confounding effects of glucose-lowering medication, established insulin resistance, severe obesity or chronic disease management. It also provides a cleaner test of whether the interruption itself, rather than disease treatment or weight-loss context, changes metabolic handling. If a low-burden interruption strategy produces measurable acute metabolic signals before overt disease appears, it may help clarify how daily sitting patterns contribute to early metabolic risk.

The practical appeal of sitting interruption is that it targets this temporal pattern directly. Short bouts of standing, walking, stair climbing, resistance activity, yoga or other light-intensity movement can be inserted into work and domestic routines without requiring a formal exercise session. Previous systematic reviews and meta-analyses have established an important foundation for this field, showing that interrupting prolonged sitting can acutely influence postprandial glucose and insulin responses [8,9,10,11,12,13]. The emerging literature on exercise snacks similarly suggests that brief activity exposures can influence postprandial glucose-insulin dynamics, particularly in metabolically vulnerable groups [14]. However, the existing evidence also exposes a key translational problem: current advice often collapses distinct prescriptions into a broad message that any movement is helpful, whereas the physiological response may depend on how often sitting is interrupted, what muscles are recruited, how intense the break is and how long postprandial metabolism is monitored.

Several limitations in previous syntheses restrict the precision of this advice. First, intervention reviews aimed at reducing sedentary time show that behavioral programs can displace some sitting, but they do not necessarily identify which acute interruption prescriptions produce the most favorable metabolic response [15,16,17]. Second, causal-inference and umbrella-review work emphasizes the breadth of sedentary-health associations but leaves open the question of which experimentally manipulated interruption features are responsible for acute metabolic change [15,18]. Third, many previous meta-analyses synthesized broad adult populations or metabolically vulnerable groups, including individuals with obesity, diabetes or older age, which limits direct inference for apparently healthy participants [10,11,13]. Fourth, prior work has often focused mainly on standing or light-intensity walking breaks, postprandial glucose and insulin, or network comparisons of interruption strategies, whereas triglycerides and less commonly reported metabolic markers such as non-esterified fatty acids (NEFAs) and C-peptide have been less consistently examined [12]. Fifth, many acute crossover trials report multiple postprandial time points, multiple intervention arms or multiple outcome definitions from the same participants. Treating all extracted effects as independent may overstate precision, whereas selecting or collapsing effect sizes may discard biologically meaningful repeated-measure information. The most directly comparable recent meta-analysis in healthy adults provided valuable evidence on postprandial metabolism, but it relied on a conventional two-level synthesis and did not fully model the dependency structure generated by repeated sampling, multiple outcomes and multiple interruption conditions within the same study [19]. Notably, our evidence base overlaps only partly with that review: of its 17 trials, 11 are also included here, while the present synthesis draws on 14 further trials not captured previously. A substantial part of the present evidence base is therefore newly incorporated or re-analyzed under a three-level dependency structure rather than reproduced with a different model. Beyond this difference in evidence base, the reliance of prior syntheses on models that do not fully capture within-study dependency makes it difficult to distinguish a robust sitting-interruption effect from a protocol-specific finding and leaves uncertainty regarding whether participant characteristics, experimental context and protocol-level factors, such as interruption modality, intensity, frequency and duration, explain variation in acute glucose, insulin and lipid responses.

The present review was therefore designed not as a simple update of previous pooled estimates but as a prescription-focused synthesis of acute metabolic responses in apparently healthy participants. We used a three-level meta-analytic framework to retain dependent effect sizes while accounting for clustering within studies, thereby preserving repeated postprandial information rather than reducing each study to a single estimate. We then evaluated glucose, insulin, triglycerides, NEFA and C-peptide together and combined this model with subgroup and meta-regression analyses of interruption modality, intensity, frequency, duration and experimental context, plus formal risk-of-bias and certainty assessments. The aim was to move beyond the broad claim that every movement counts and toward a more cautious and metabolically specific question: which sitting interruptions show acute metabolic signals, under what protocol conditions, and for which endpoints?

## 2. Materials and Methods

This three-level meta-analysis was conducted in accordance with the Preferred Reporting Items for Systematic Reviews and Meta-Analyses (PRISMA) 2020 statement [20] (Supplementary Table S1) and was prospectively registered with the International Prospective Register of Systematic Reviews (PROSPERO; CRD420261333108).

### 2.1. Information Sources

We systematically searched PubMed (NCBI), Web of Science Core Collection, Cochrane Library, Embase, and Scopus from database inception through March 2026. Eligible records were restricted to full-text, peer-reviewed journal articles published in English. No restrictions were imposed on publication date or sample size.

### 2.2. Search Strategy

The search strategy, structured around the PICOS framework, targeted two core concept clusters: (i) intervention-related terms (“sedentary behavior,” “sitting,” “prolonged sedentary behavior,” “interruption,” “sedentary interruption”); and (ii) outcome-related terms encompassing glucose metabolism, lipid metabolism and cardiometabolic health. All databases were searched using free-text terms only, applied to the title, abstract, and (where available) keyword fields; database-specific controlled vocabulary (e.g., MeSH in PubMed and the Cochrane Library, Emtree in Embase) was not used. This kept the search syntax uniform across the five databases—two of which (Web of Science Core Collection and Scopus) do not provide a controlled indexing thesaurus—while prioritizing a highly sensitive, keyword-based strategy supplemented by extensive snowballing. The exact field tags used in each database (Title/Abstract in PubMed, :ti,ab in Embase, :ti,ab,kw in the Cochrane Library, Topic [TS] in Web of Science, and TITLE-ABS-KEY in Scopus) and the complete search strings are reported in Supplementary Table S2. To maximize sensitivity, no population-restrictive filters were applied. Three complementary snowball strategies were implemented: (i) manual screening of reference lists of all included studies; (ii) forward citation tracking of included studies; and (iii) related-articles searches in MEDLINE and Embase. We additionally screened articles identified in prior systematic reviews of sedentary interruption and health outcomes. PROSPERO and the Cochrane Database of Systematic Reviews were searched to verify whether a relevant registered or published protocol existed. The complete PubMed Boolean search string is provided in Supplementary Table S2.

### 2.3. Eligibility Criteria

Inclusion and exclusion criteria were defined according to the PICOS framework. Studies were eligible if they simultaneously satisfied all of the following conditions. Population (P): apparently healthy individuals free of diagnosed cardiometabolic disease or other acute or chronic conditions known to alter glucose, insulin, or lipid metabolism; participants were required to be free of diabetes, cardiovascular disease, hypertension, metabolic syndrome, and severe hepatic, renal, or endocrine disorders, and not be taking medications known to influence glucolipid metabolism. This population was selected deliberately: restricting inclusion to apparently healthy individuals isolates the acute metabolic response to sitting interruption itself, free from the confounding influence of glucose-lowering medication, established insulin resistance or ongoing disease management, and allows the earliest, potentially reversible stage of metabolic strain to be examined before overt cardiometabolic disease develops. Intervention (I): any planned or structured interruption of prolonged sitting, including standing and low-, moderate- or high-intensity physical activity such as walking, cycling, running, stair climbing, resistance activity or mind–body activity. Comparison (C): continuous, uninterrupted sitting on the same experimental day, with only essential, brief postural breaks (e.g., restroom visits) permitted if explicitly documented. Outcomes (O): at least one acute glucose or lipid metabolism outcome. Glucose metabolism outcomes comprised the incremental area under the curve (iAUC), total area under the curve (AUC), fasting or postprandial blood glucose, fasting or postprandial insulin, indices of insulin resistance, and time in hyperglycemia. The primary lipid outcome was fasting or postprandial triglycerides (TG), selected because it is the most sensitive marker of acute postprandial lipid metabolism and is most consistently reported in single-day sitting-interruption protocols. NEFA and C-peptide were treated as exploratory secondary outcomes rather than primary endpoints. Study design (S): eligible studies were acute randomized crossover trials conducted under controlled Laboratory or Simulated real-world experimental conditions, with experimental conditions performed on separate study visits and separated by an inter-condition interval intended to minimize carryover effects.

Studies were excluded if they (1) lacked a proper control condition (e.g., single-arm or uncontrolled designs); (2) did not report glucose or lipid metabolism outcomes; (3) investigated long-term exercise training rather than acute responses; (4) conducted crossover conditions without an inter-condition separation period; (5) administered medications or special diets during the experimental period; or (6) were qualitative studies, uncontrolled free-living interventions, observational studies, systematic reviews or meta-analyses, study protocols, gray literature, or abstract-only publications.

### 2.4. Study Selection

**Figure 1:**
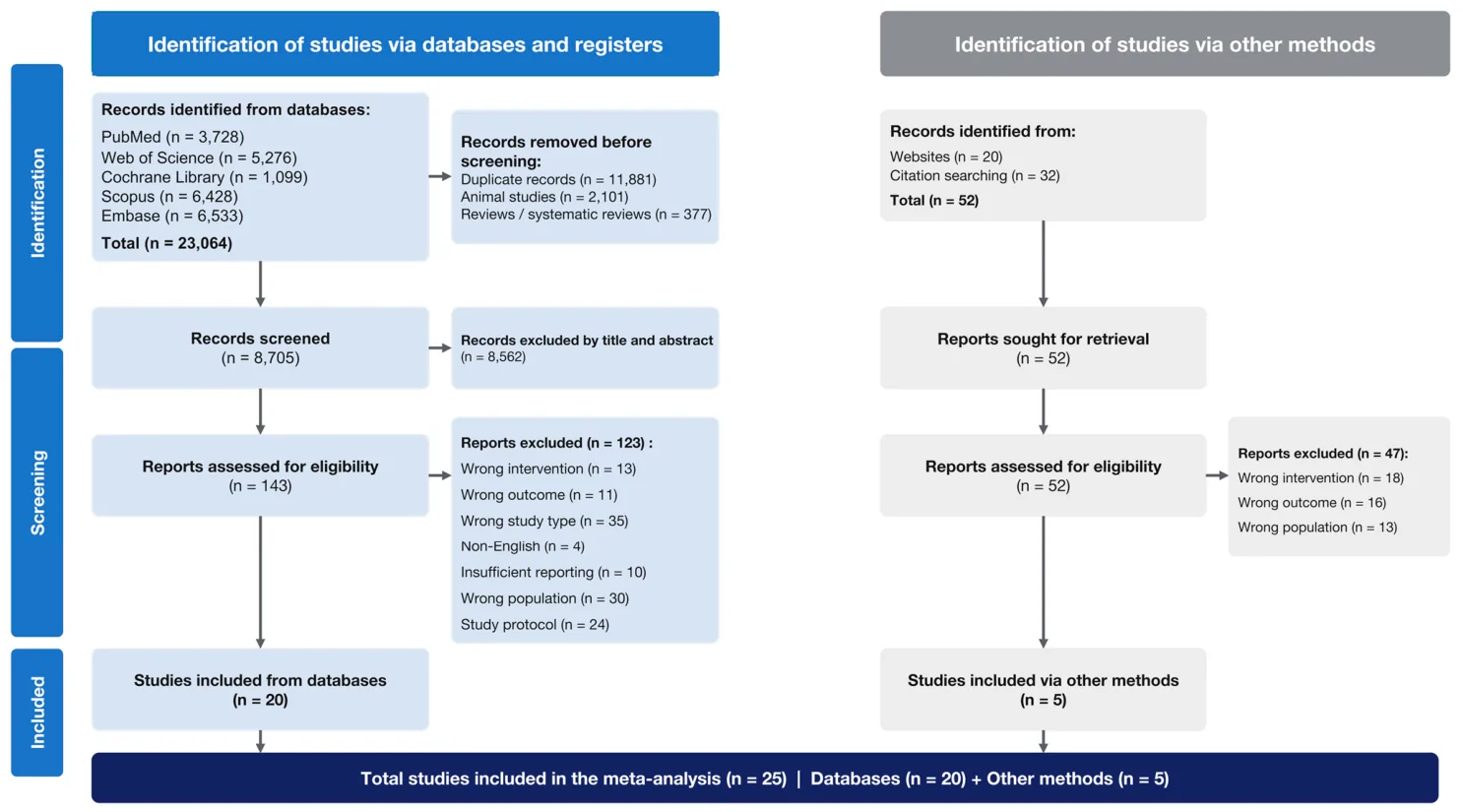
PRISMA flow diagram for study identification, screening, eligibility assessment and inclusion.

After deduplication in EndNote (version X9), two independent investigators screened records against the eligibility criteria using Zotero (version 7.0.22). Discrepancies were resolved through discussion or adjudication by a third investigator. The same two investigators then independently reviewed the full texts of potentially eligible articles, with disagreements resolved via the same adjudication procedure. The study-selection flow is shown in Figure 1. Formal inter-rater agreement statistics were not calculated for either title/abstract or full-text screening because item-level pre-consensus decisions were not retained after discrepancies had been reconciled. Consequently, neither Cohen’s κ nor percentage agreement could be calculated retrospectively.

### 2.5. Data Extraction and Coding

Two investigators independently extracted data using a standardized extraction form. Before formal extraction, this form was pilot-tested by both investigators on a random subset of five included studies to verify that all predefined fields (study characteristics, participant characteristics, sitting-interruption protocol characteristics, and outcome data) were clearly defined, unambiguous, and consistently interpretable. Discrepancies identified during piloting were discussed, and the form was refined accordingly, including clarification of field definitions and the handling of multi-arm and multi-time-point data, before it was applied to the full set of studies. Discrepancies during formal extraction were resolved by a third investigator. Formal inter-rater agreement statistics were not calculated for data extraction because item-level pre-consensus extraction records were not retained after reconciliation; consequently, neither Cohen’s κ nor percentage agreement could be calculated retrospectively. All extraction discrepancies were resolved through discussion, with third-investigator adjudication when necessary. Extracted information comprised (i) study characteristics (first author, publication year and country of origin); (ii) participant characteristics (sample size, age, sex and body mass index [BMI]); and (iii) sitting-interruption protocol characteristics (modality, setting, frequency, duration per break, total number of breaks and total experimental day duration). Additional crossover-specific information included the reported interval between experimental conditions, whether experimental conditions were conducted on separate study visits, sequence balancing or randomization, and any statistical adjustment for period or order effects. Study and protocol characteristics are summarized in Supplementary Table S3. When numerical data were missing or presented exclusively in graphical form, corresponding authors were contacted. If no response was received and figures were of sufficient resolution, data were extracted using WebPlotDigitizer (version 4.1; https://automeris.io/WebPlotDigitizer (accessed on 13 June 2026)). Studies for which critical data remained unobtainable were excluded.

### 2.6. Risk of Bias and Crossover-Specific Carryover Assessment

Risk of bias was assessed using the Cochrane Risk of Bias 2 (RoB 2) tool [21], which evaluates five domains: (1) bias arising from the randomization process; (2) bias due to deviations from intended interventions; (3) bias due to missing outcome data; (4) bias in measurement of the outcome; and (5) bias in selection of the reported result. Domain-level judgments were synthesized to yield an overall risk-of-bias rating for each study: low risk, some concerns or high risk. Two reviewers performed independent assessments; discrepancies were resolved by a third reviewer. Domain-level and overall judgments are reported in Supplementary Figure S1 and Supplementary Table S4. Given that all included studies used crossover designs, the RoB 2 assessment was supplemented by a dedicated evaluation of washout adequacy and potential carryover risk. This evaluation considered the reported interval between experimental conditions, whether experimental conditions were conducted on separate study visits, and any reported balancing or statistical adjustment for sequence or order effects. For these acute, single-day protocols, reported inter-condition intervals of at least 72 h were operationally categorized as adequate; intervals permitting a minimum separation of only 24 h were classified as raising potential concern, and separate visits for which the exact inter-visit interval was not reported were classified as unclear. Full study-level judgments are provided in Supplementary Table S8.

### 2.7. Statistical Analysis

All analyses were performed in R (version 4.4.1; R Foundation for Statistical Computing, Vienna, Austria). Three-level meta-analytic models were fitted using the metafor package (version 4.6-0); cluster-robust variance estimation (CRVE) with CR2 small-sample correction was implemented via the clubSandwich package (version 0.5.11). A two-sided *p* < 0.05 was considered statistically significant unless otherwise stated. Detailed subgroup estimates are provided in Supplementary Table S5.

#### 2.7.1. Effect Size Calculation and Three-Level Meta-Analysis

Primary studies investigating the metabolic effects of sitting interruption typically report multiple correlated outcomes—iAUC, total AUC, standard AUC, and mean glucose—at multiple postprandial time points within the same experimental day. A single study therefore generates numerous dependent effect sizes. Treating these as independent in pooled analyses violates a core assumption of conventional meta-analysis; conversely, selecting a single outcome or retaining only one effect size per study is overly conservative and discards meaningful information. We addressed this by fitting a three-level random-effects meta-analytic model that partitions total variance into sampling variance (Level 1), within-cohort effect-size variance (Level 2), and between-cohort variance (Level 3) [22,23]. This framework explicitly models the hierarchical data structure and dependency among effect sizes while retaining all available outcomes, thereby preserving statistical power and estimation precision. Reports were screened for potential participant overlap using authorship, recruitment setting and dates, sample characteristics, intervention protocols, trial-registration numbers and ethics identifiers. Ma et al. [24,25] (2020/2021; ChiCTR1800018829) and Engeroff et al. [26,27] (2017/2022; ethics approval no. 121/13) were confirmed as two sets of companion publications; non-duplicated outcome data were retained, but each publication pair was assigned a common cohort identifier. Accordingly, effect sizes were nested within independent cohorts in the three-level random-effects model, and CR2 robust inference was clustered at the independent cohort rather than the publication level.

To strengthen inference under effect-size dependency, CRVE with a working variance–covariance matrix was incorporated into the three-level model using the CR2 small-sample correction, yielding robust standard errors even when the number of clusters was limited [28]. Because the primary trials did not report the correlations among their repeated within-study effect sizes, these correlations could not be estimated directly and were instead imputed. For the primary analysis, a constant within-study correlation of ρ = 0.6 was assumed for all dependent effect sizes—a moderate value chosen because repeated postprandial measures within the same participants are expected to be substantially but not perfectly correlated—and its influence was formally examined in sensitivity analyses (Section 2.7.3). A key strength of combining this working correlation with CR2 cluster-robust variance estimation is that the resulting inference remains valid even if the assumed value departs from the true within-study correlation. A random-effects specification was chosen to accommodate true effect heterogeneity across studies. Variance components were estimated via restricted maximum likelihood (REML); maximum likelihood (ML) estimation was performed in parallel as a sensitivity check. This three-level formulation was preferred over two alternative strategies for handling dependent effect sizes. A multivariate meta-analysis would explicitly model the correlations among outcomes, but it requires the full within-study covariance matrix among effect sizes, which was not reported by the primary trials and could not be reliably reconstructed from the available data. A conventional single- or two-level model would instead either ignore the within-study dependency or impose a restrictive assumption about its correlation structure. By contrast, the three-level random-effects model combined with cluster-robust variance estimation absorbs this dependency through the hierarchical random-effects structure while remaining robust to misspecification of the exact within-cluster correlation and does so without requiring the unavailable covariance matrix. This combination therefore offered the most defensible balance between retaining all repeated-measure information and protecting inference against unknown dependency [22,23,28].

Effect sizes were expressed as standardized mean differences (SMD) with Hedges’ *g* correction for small-sample bias and interpreted using conventional thresholds: trivial (*g* < 0.2), small (0.2 ≤ *g* < 0.5), moderate (0.5 ≤ *g* < 0.8), and large (*g* ≥ 0.8). Pooled effect sizes are reported with 95% confidence intervals (CI). Prediction intervals (PI) were computed from the t-distribution to estimate the plausible range of true effects in future comparable studies. Heterogeneity was assessed using Cochrane’s Q, I^2^, and τ^2^, with within-cohort (Level 2) and between-cohort (Level 3) variance components reported separately to localize sources of heterogeneity. As a supplementary analysis, the statistical power of the primary pooled estimates was evaluated using the metameta package to gauge the risk of false-negative findings given the observed sample size and heterogeneity; these results were used for auxiliary interpretation only. For NEFA and C-peptide, informed by only three and two independent cohorts, respectively, the prespecified models were fitted for consistency; however, the three-level variance structures could not be identified reliably. Their point estimates were therefore treated as simple descriptive pooled effects without confirmatory hierarchical inference.

#### 2.7.2. Subgroup Analyses and Meta-Regression

Subgroup analyses were organized by prespecified moderator families when outcome-level data were sufficient for exploratory comparison. Subgroup-specific estimates are reported to describe the available evidence, but categories supported by fewer than five independent cohorts were interpreted descriptively rather than confirmatorily; formal inference was emphasized at the moderator-family level. Meta-regression was conducted for continuous moderators when at least 10 effect sizes were available and the cluster structure permitted stable model estimation. Predefined categorical moderators included participant age group (children, 4–9 years; adolescents, 10–17 years; young adults, 18–35 years; middle-aged adults, 36–64 years; older adults, 65 years or older), outcome metric (e.g., iAUC, AUC, mean glucose), interruption modality (standing, walking, stair climbing, resistance exercise, mind–body exercise, cycling and running), exercise intensity (low, moderate or high, classified according to American College of Sports Medicine criteria where sufficient intensity information was available), and experimental setting (Laboratory or Simulated). For moderator analyses, the Simulated category combined studies described in Table 1 as “Simulated setting” and “Simulated real-world setting”, whereas studies described as “Laboratory” were retained in the Laboratory category. Reported intensity indicators included heart rate reserve (HRR) and rating of perceived exertion (RPE), where available. The children and adolescent age categories were prespecified a priori for completeness of the moderator framework; consistent with the adult-only eligibility criteria, no pediatric trials were expected or identified, and these two categories therefore contributed no data to any analysis. Continuous moderators examined via meta-regression included age, BMI, proportion of female participants, break frequency, number of breaks, duration per break, and total experimental exposure time. Meta-regression results were visualized using ggplot2. Given the limited number of studies available for several moderators, all subgroup and meta-regression analyses were regarded as exploratory and hypothesis-generating rather than as confirmatory tests and were interpreted accordingly throughout. Washout adequacy was not examined as a moderator because only two independent cohorts were classified as raising potential concern and one as unclear, resulting in highly imbalanced categories and insufficient independent-cohort support for meaningful between-category inference.

#### 2.7.3. Publication Bias and Sensitivity Analyses

Publication bias was assessed through visual inspection of funnel plots and Egger’s test when ≥10 studies were available (*p* > 0.05 indicating no significant asymmetry). Both subjective appraisal and statistical testing informed the overall judgment regarding small-study effects. Sensitivity analyses included varying the imputed within-study correlation around its primary value (ρ = 0.4 and ρ = 0.8, versus 0.6 in the primary analysis) and iterative leave-one-out analysis to identify individual studies exerting disproportionate influence on the pooled estimates.

### 2.8. Certainty of Evidence

Two investigators independently rated the certainty of evidence for each outcome using the Grading of Recommendations Assessment, Development, and Evaluation (GRADE) framework [50], classifying evidence as high, moderate, low or very low. Baseline certainty was downgraded according to the following pre-specified criteria: (1) risk of bias, downgraded one level for some concerns or two levels for predominantly high risk across included studies; (2) inconsistency, downgraded according to heterogeneity and consistency of effect direction; (3) imprecision, downgraded when the pooled estimate was non-significant or had a wide confidence interval; and (4) publication bias, downgraded when Egger’s test was significant (*p* < 0.05). Discrepancies were resolved through consensus or adjudication by a third investigator. The GRADE evidence profile is shown in Supplementary Table S6.

## 3. Results

### 3.1. Search Results

As shown in Figure 1, the database search identified a total of 23,064 records (PubMed, n = 3728; Web of Science, n = 5276; Cochrane Library, n = 1099; Scopus, n = 6428; and Embase, n = 6533). Before screening, 14,359 records were removed, leaving 8705 records for title and abstract screening. Of these, 8562 were excluded, and 143 reports proceeded to full-text assessment. During full-text screening, 123 reports were excluded, resulting in the inclusion of 20 studies identified through database searches. An additional 52 records were identified through other sources, including websites (n = 20) and citation searching (n = 32). After full-text assessment, 47 reports were excluded, resulting in the inclusion of 5 additional studies. In total, 25 publications representing 23 independent cohorts were included in this systematic review and meta-analysis.

### 3.2. Study Selection and Evidence Base

The final evidence base comprised randomized crossover trials conducted in apparently healthy participants under controlled Laboratory or Simulated free-living conditions [24,25,26,27,29,30,31,32,33,34,35,36,37,38,39,40,41,42,43,44,45,46,47,48,49]. Study and protocol characteristics, including participant age, sex distribution, BMI, experimental setting, interruption modality, break frequency, break duration and total experimental exposure time, are summarized in Table 1, with full extraction details in Supplementary Table S3. Risk-of-bias judgments were mainly driven by limitations intrinsic to acute behavioral crossover trials, particularly incomplete blinding and incomplete reporting of randomization or selective-reporting safeguards; because all outcomes were objective biochemical measures, the practical impact of incomplete participant blinding on the effect estimates is expected to be limited (see Section 4). The full domain-level profile is shown in Supplementary Figure S1 and Supplementary Table S4. The crossover-specific assessment indicated that 20 of the 23 independent cohorts, represented by 22 publications, reported inter-condition intervals of at least 72 h and were judged to have adequate washout. Two cohorts, Kuo et al. [32] (2024) and Chueh et al. [36] (2025), permitted intervals as short as 24 h and were classified as raising potential concern. Colvin et al. [37] (2024) conducted the experimental conditions on separate study visits, confirming the presence of an inter-visit interval but did not report its exact duration; carryover risk was therefore classified as unclear. No included cohort was conducted without separation between experimental conditions. Full study-level details are provided in Supplementary Table S8.

### 3.3. Overall Pooled Effects

Across the outcome-specific analyses, the dataset included 135 glucose effect sizes from 23 independent cohorts, 49 insulin effect sizes from 12 cohorts, 60 triglyceride effect sizes from 12 cohorts, 47 non-esterified fatty acid (NEFA) effect sizes from three cohorts and 23 C-peptide effect sizes from two cohorts. Negative Hedges’ *g* values indicate lower metabolic responses during sitting-interruption conditions than during uninterrupted sitting.

**Figure 2:**
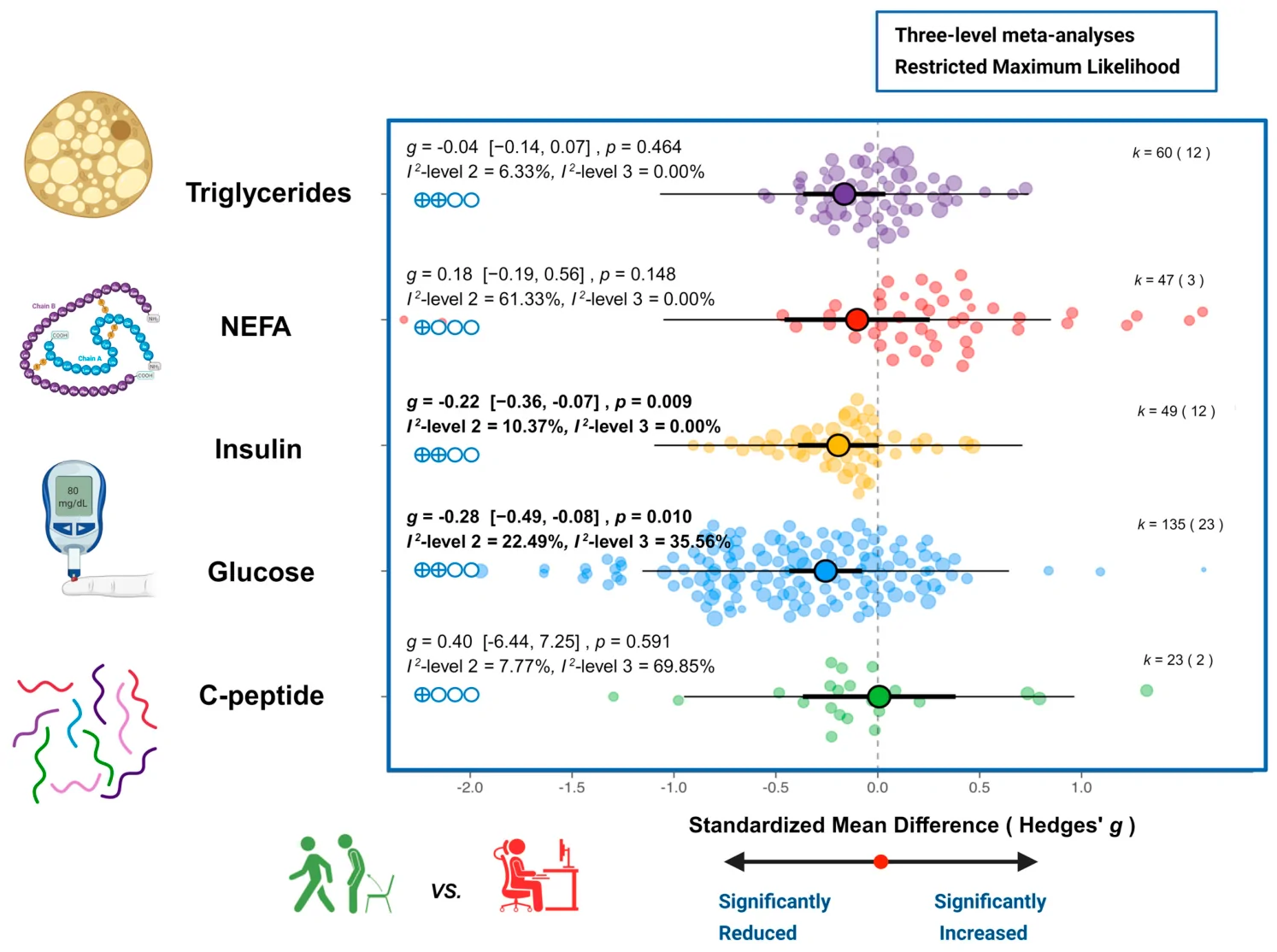
Overall pooled effects of sitting interruption on glucose and lipid metabolic outcomes. Outcome-specific estimates were obtained from three-level random-effects meta-analyses fitted using restricted maximum likelihood, with cluster-robust variance estimation and the CR2 small-sample correction applied at the independent-cohort level. Small translucent points indicate individual effect sizes, larger filled circles indicate pooled Hedges’ *g* estimates, and horizontal bars indicate 95% confidence intervals. Negative values indicate lower metabolic responses under sitting-interruption conditions than under uninterrupted sitting. Text within each row reports Hedges’ *g*, the 95% confidence interval, *p* value, Level-2 and Level-3 I^2^ statistics, and the number of effect sizes and independent cohorts, presented as *k* = effect sizes (independent cohorts). GRADE certainty symbols are displayed beside each outcome. Estimates for non-esterified fatty acids (NEFA) and C-peptide are descriptive only; with three and two independent cohorts, respectively, hierarchical variance was not reliably estimable, and inferential statistics are presented for transparency rather than confirmatory inference.

Sitting interruption was associated with small pooled reductions in glucose (Hedges’ *g* = −0.28, 95% CI −0.49 to −0.08, *p* = 0.010) and insulin (*g* = −0.22, 95% CI −0.36 to −0.07, *p* = 0.009; Figure 2). Both estimates are small in magnitude, corresponding to less than one-third of a standard deviation and falling at the lower end of the conventional small-effect range. Their statistical significance therefore reflects the consistency of a small effect across many effect sizes rather than a large or clinically decisive response and should be interpreted together with the broad prediction intervals and low certainty of evidence. The overall triglyceride effect was not significant (*g* = −0.04, 95% CI −0.14 to 0.07, *p* = 0.464). For the exploratory secondary outcomes, the prespecified three-level models yielded NEFA estimates based on 47 effect sizes from three independent cohorts (*g* = 0.18, 95% CI −0.19 to 0.56, *p* = 0.148) and C-peptide estimates based on 23 effect sizes from two independent cohorts (*g* = 0.40, 95% CI −6.44 to 7.25, *p* = 0.591). However, with only three and two independent cohorts, respectively, the hierarchical variance structures could not be identified reliably. The confidence intervals and *p* values are therefore reported for transparency but are highly unstable, and the point estimates should be interpreted only as descriptive pooled effects rather than as confirmatory evidence supported by a validated three-level structure. The prediction interval for glucose was broad, indicating substantial between-protocol uncertainty. Certainty of evidence was low for glucose, insulin and triglycerides, and very low for NEFA and C-peptide (Table 2; detailed GRADE profile in Supplementary Table S6).

### 3.4. Subgroup Analyses

**Figure 3:**
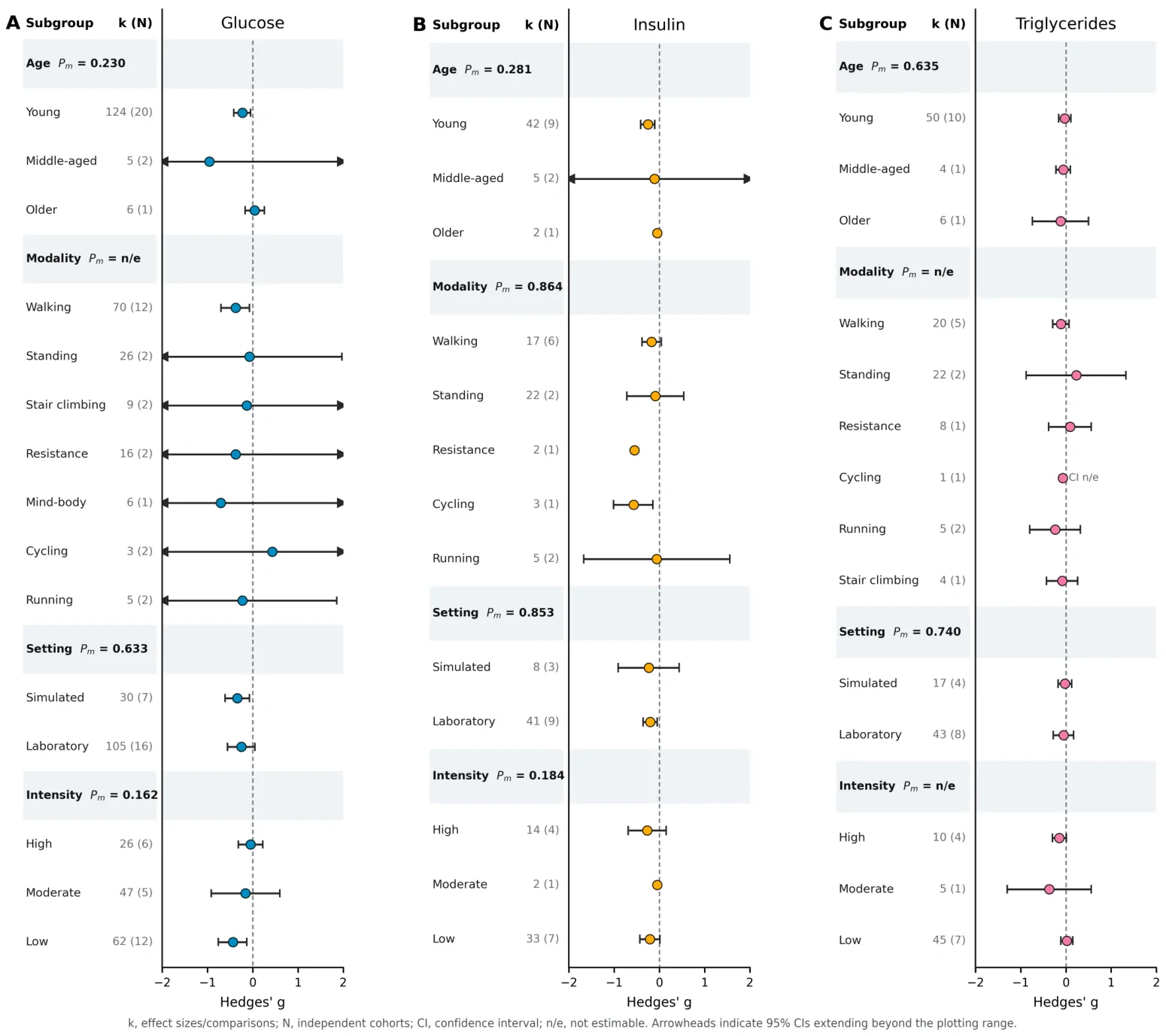
Subgroup analyses for glucose (**A**), insulin (**B**) and triglycerides (**C**). Points indicate subgroup-specific Hedges’ *g* estimates and horizontal bars indicate 95% confidence intervals; arrowheads denote intervals extending beyond the plotting range. *Pm* is the *p* value for the between-subgroup (moderator) test, estimated with cluster-robust variance at the cohort level. No moderator reached statistical significance for any outcome. Estimates based on fewer than five independent cohorts are exploratory and hypothesis-generating only. *k*, effect sizes/comparisons; *N*, independent cohorts; CI, confidence interval; n/e, not estimable (moderator model did not converge because one or more categories contained fewer than two independent cohorts). Detailed numerical estimates are provided in Supplementary Table S5. For the setting moderator, “Simulated” combines the “Simulated setting” and “Simulated real-world setting” descriptors reported in Table 1.

Subgroup analyses for the three primary outcomes are shown in Figure 3, with detailed estimates in Supplementary Table S5. For glucose, age did not significantly modify the pooled effect (*Pm* = 0.23). A significant within-subgroup reduction was observed in young adults (*g* = −0.23, 95% CI −0.42 to −0.05, *p* = 0.016; 124 effect sizes from 20 cohorts). However, this within-subgroup finding should not be interpreted as evidence of age-related effect modification because the between-age moderator test was not statistically significant. Estimates for middle-aged adults (five effect sizes, two cohorts) and older adults (six effect sizes, one cohort) were based on fewer than five independent cohorts and are therefore exploratory and hypothesis-generating only. The between-modality moderator test could not be estimated under cohort-level cluster-robust variance estimation because of sparse categories. Within the walking subgroup, sitting interruption was associated with a reduction in glucose (*g* = −0.38, 95% CI −0.70 to −0.07, *p* = 0.022; 70 effect sizes from 12 cohorts); however, this within-subgroup estimate does not establish that walking is superior to other interruption modalities. All remaining modality categories, including stair climbing, resistance activity, cycling and running, were informed by fewer than five independent cohorts and are therefore exploratory and hypothesis-generating. Neither setting (*Pm* = 0.63) nor intensity (*Pm* = 0.16) significantly modified glucose responses. Accordingly, the available evidence does not support ranking interruption settings, modalities or intensity levels.

For insulin, age (*Pm* = 0.28), modality (*Pm* = 0.86), setting (*Pm* = 0.85) and intensity (*Pm* = 0.18) did not significantly modify the pooled effect (Figure 3). Young adults showed a significant within-subgroup reduction (*g* = −0.25, 95% CI −0.41 to −0.10, *p* = 0.007; 42 effect sizes from nine cohorts). All modality and intensity categories other than walking were drawn from fewer than five independent cohorts and are reported as exploratory and hypothesis-generating; the larger resistance-exercise estimate was based on only two effect sizes from a single cohort and should be interpreted descriptively rather than prescriptively.

For triglycerides, neither age (*Pm* = 0.64) nor experimental setting (*Pm* = 0.74) significantly modified the pooled effect (Figure 3). The between-modality and intensity moderator tests could not be estimated under cohort-level cluster-robust variance estimation because of sparse subgroup cells. No subgroup-specific estimate reached statistical significance; in particular, the moderate-intensity estimate, derived from five effect sizes within a single independent cohort, was considered exploratory and hypothesis-generating rather than evidence of a subgroup effect.

### 3.5. Meta-Regression

**Figure 4:**
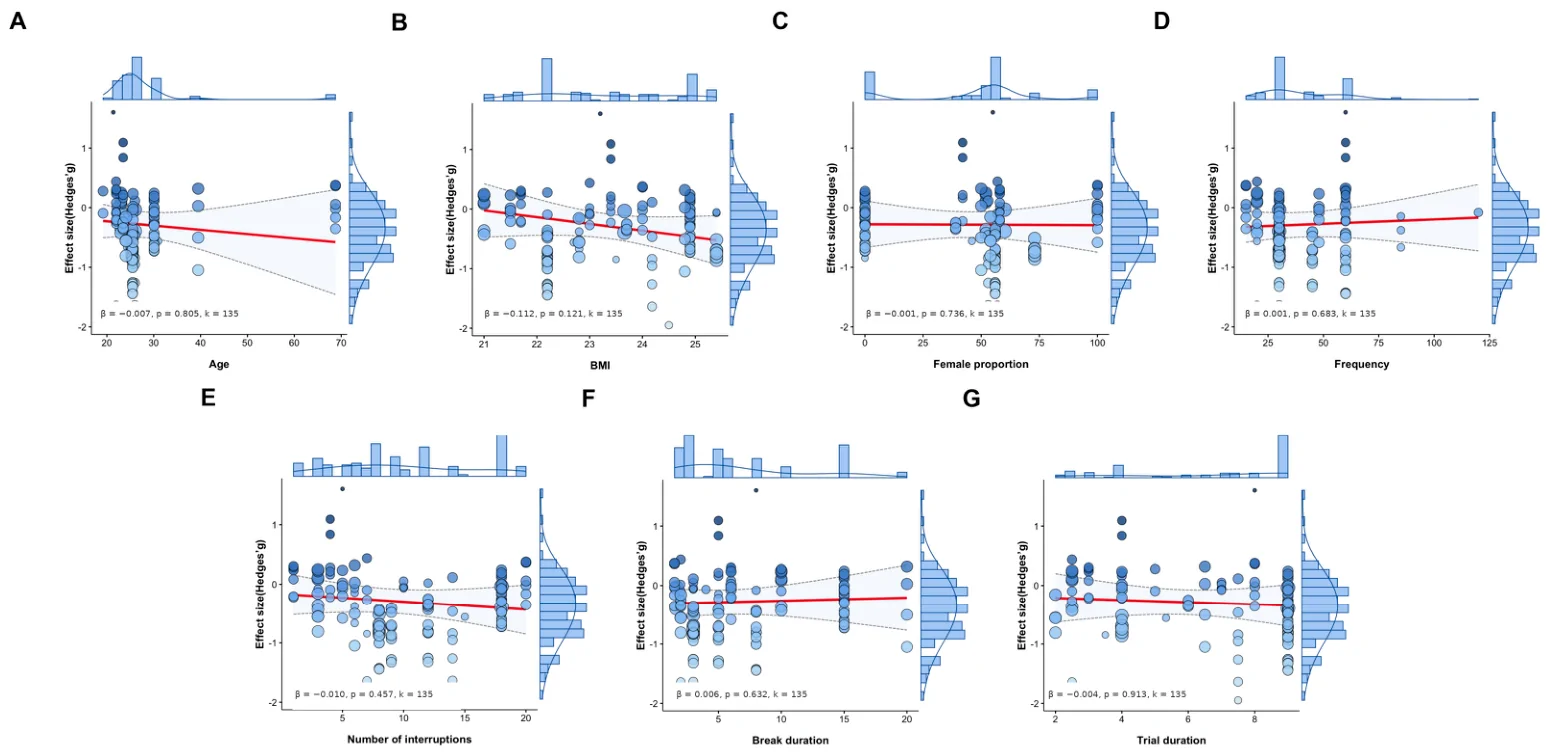
Meta-regression analyses for glucose. Each panel shows the meta-regression of the glucose effect size on one continuous moderator: (**A**) age; (**B**) body mass index; (**C**) female proportion; (**D**) break frequency (minutes between interruptions); (**E**) number of interruptions; (**F**) break duration; (**G**) total experimental exposure time. Bubble sizes reflect relative precision; fitted lines summarize study-level linear associations; dashed bands indicate confidence bands.

**Figure 5:**
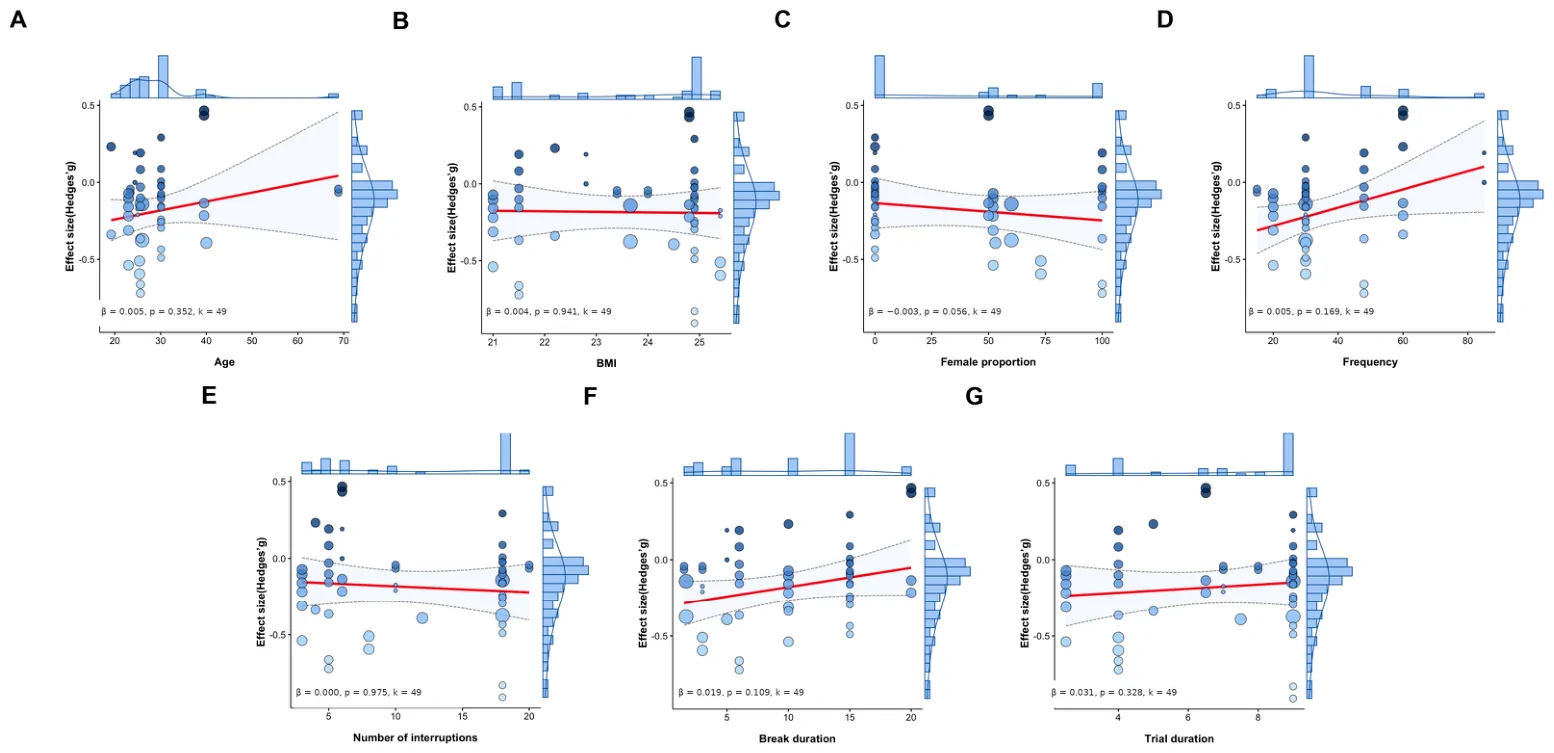
Meta-regression analyses for insulin. Each panel shows the meta-regression of the insulin effect size on one continuous moderator: (**A**) age; (**B**) body mass index; (**C**) female proportion; (**D**) break frequency (minutes between interruptions); (**E**) number of interruptions; (**F**) break duration; (**G**) total experimental exposure time. Bubble sizes reflect relative precision; fitted lines summarize study-level linear associations; dashed bands indicate confidence bands.

**Figure 6:**
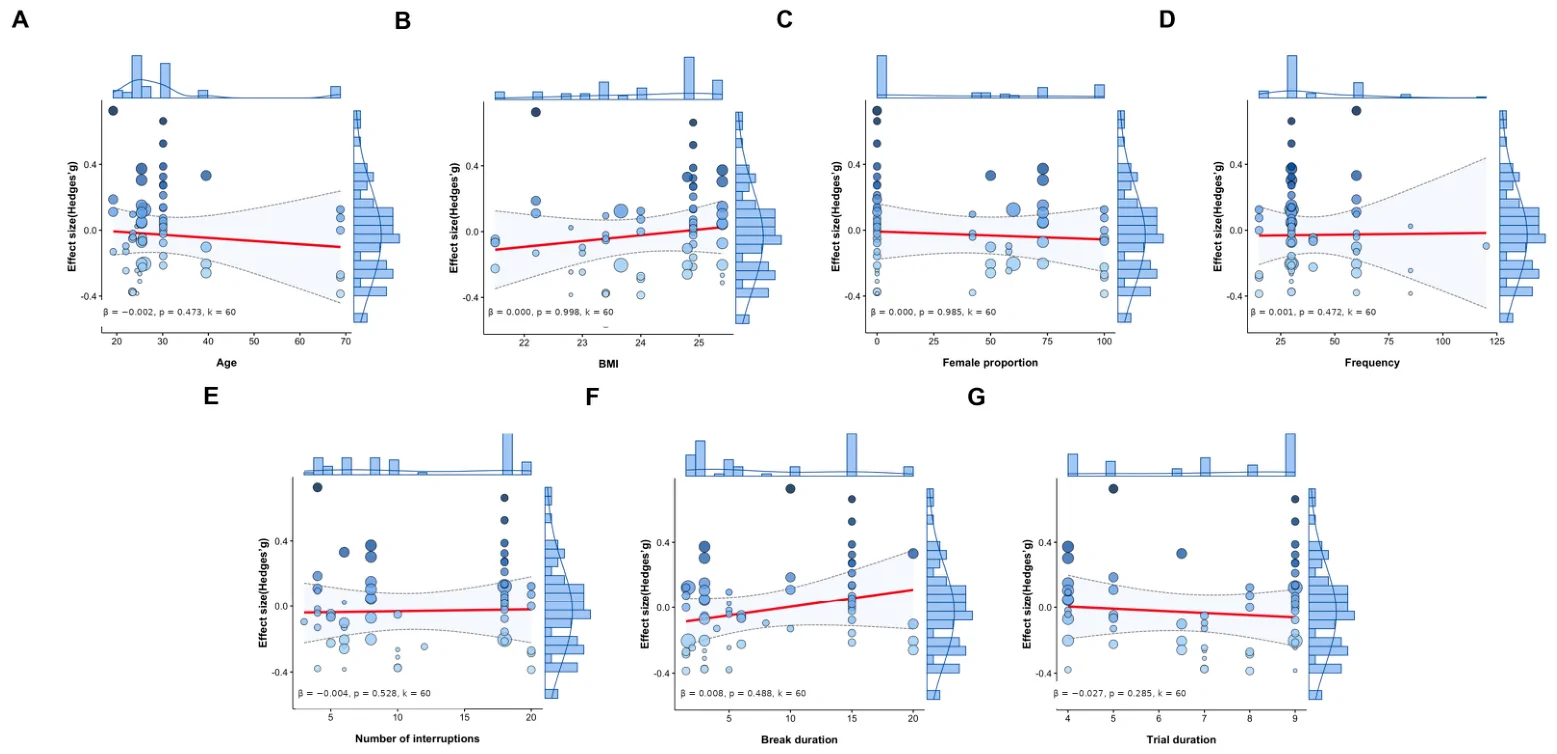
Meta-regression analyses for triglycerides. Each panel shows the meta-regression of the triglycerides effect size on one continuous moderator: (**A**) age; (**B**) body mass index; (**C**) female proportion; (**D**) break frequency (minutes between interruptions); (**E**) number of interruptions; (**F**) break duration; (**G**) total experimental exposure time. Bubble sizes reflect relative precision; fitted lines summarize study-level linear associations; dashed bands indicate confidence bands.

Meta-regression analyses identified no statistically significant linear associations between effect size and age, BMI, sex distribution, break frequency, number of breaks, break duration or total experimental exposure time for glucose, insulin or triglycerides (Figure 4, Figure 5 and Figure 6). For insulin, break frequency was coded as minutes between interruptions, with lower values indicating more frequent breaks. Break frequency was not significantly associated with insulin effect size (β = 0.005, 95% CI −0.004 to 0.014, *p* = 0.169; 49 effect sizes from 12 independent cohorts). This non-significant finding was considered exploratory only and does not establish that more frequent interruptions produce greater reductions in insulin responses. Detailed coefficients for all continuous moderators are provided in Supplementary Table S7.

### 3.6. Publication Bias, Sensitivity Analyses and Certainty of Evidence

**Figure 7:**
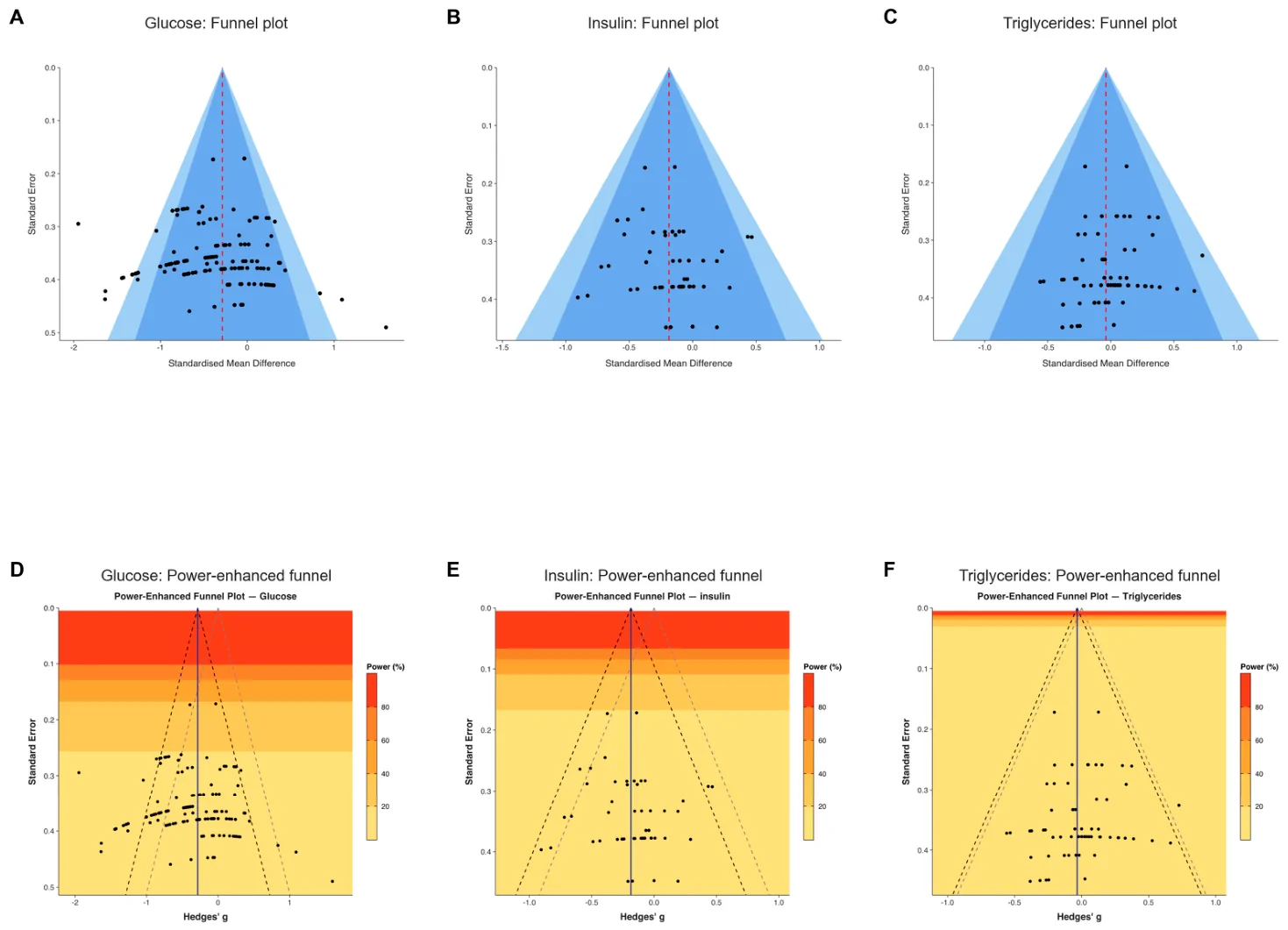
Funnel-plot diagnostics for glucose, insulin and triglycerides. Panels (**A**–**C**) show conventional funnel plots for glucose, insulin and triglycerides, respectively. Panels (**D**–**F**) show the corresponding power-enhanced funnel plots. Each point represents an individual effect size, with Hedges’ *g* displayed on the horizontal axis and the corresponding standard error on the vertical axis. In the power-enhanced funnel plots, background shading indicates estimated statistical power, with warmer and darker colors representing greater power. Funnel-plot patterns were interpreted together with the corresponding Egger’s regression tests when at least 10 independent cohorts were available.

Funnel plot inspection and Egger’s tests did not indicate significant small-study effects for glucose (z = 1.30, *p* = 0.195), insulin (z = 0.65, *p* = 0.516) or triglycerides (z = −0.73, *p* = 0.464; Figure 7). However, with only 12 to 23 independent cohorts per outcome, Egger’s regression has limited statistical power to detect funnel-plot asymmetry, so these non-significant results should be interpreted as an absence of strong evidence for small-study effects rather than as evidence that such effects are absent. The certainty of evidence was rated as low for the three primary outcomes, mainly because of risk-of-bias concerns and inconsistency; the certainty for NEFA and C-peptide was very low because the evidence base was sparse and imprecise (Supplementary Table S6).

**Figure 8:**
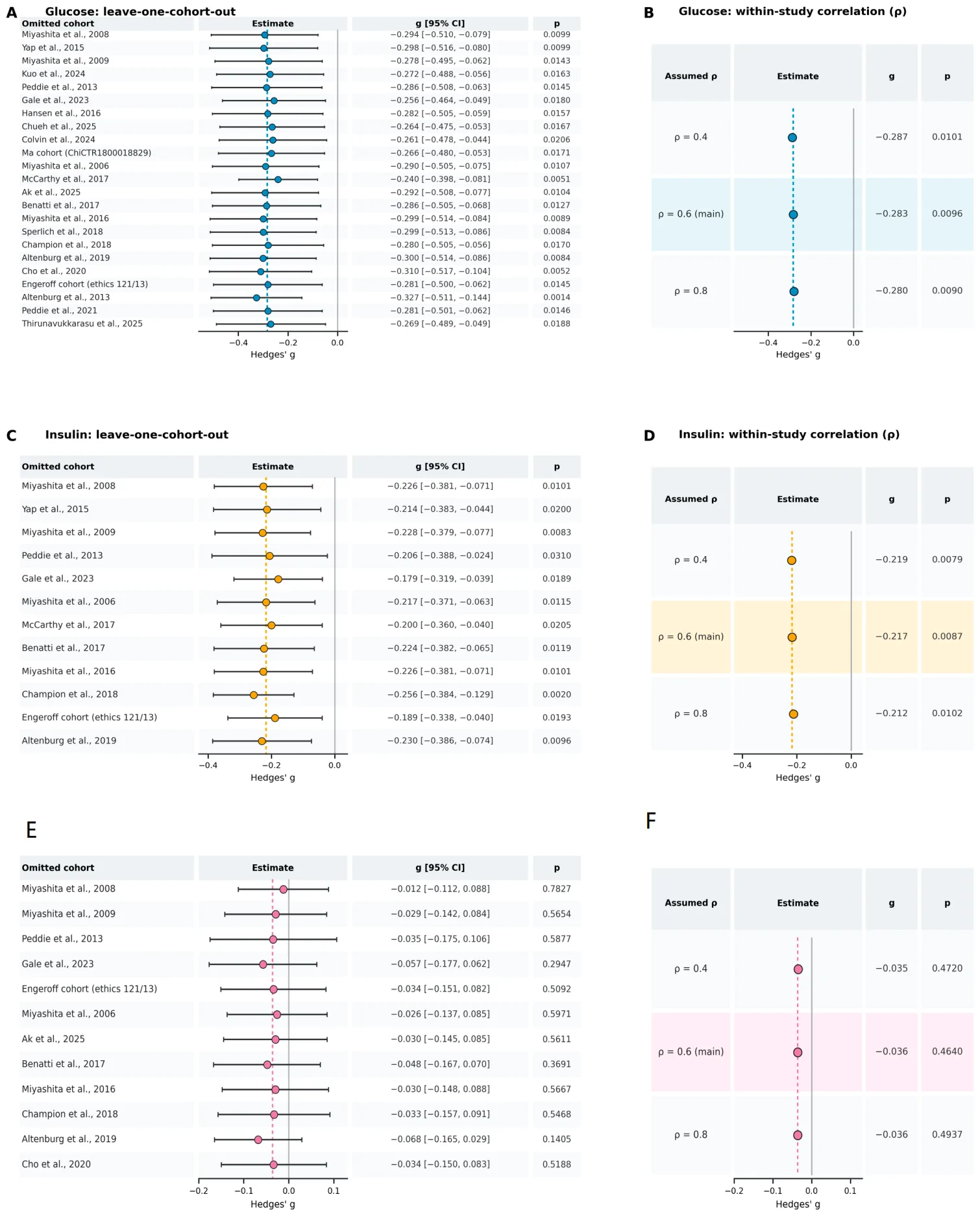
Sensitivity analyses for glucose, insulin and triglycerides. Panels (**A**,**C**,**E**) show leave-one-cohort-out analyses for glucose, insulin and triglycerides, respectively. Points represent the pooled Hedges’ *g* estimates obtained after omitting each independent cohort, and horizontal bars indicate 95% confidence intervals. Companion publications derived from the same participant cohort were omitted together as a single independent cohort. Panels (**B**,**D**,**F**) show sensitivity analyses using assumed within-study correlations of ρ = 0.4, 0.6 and 0.8, with ρ = 0.6 representing the primary analysis. Dashed colored lines indicate the corresponding full-data estimates, and solid gray lines indicate the null value. Confidence intervals are not displayed in Panels (**B**,**D**,**F**) because the correlation-sensitivity output reports only Hedges’ *g* estimates and *p* values.

Leave-one-cohort-out analyses showed that the glucose effect remained consistently negative and statistically significant after omission of each individual cohort (*g* = −0.327 to −0.240, *p* = 0.001–0.021). The insulin effect was similarly robust (*g* = −0.256 to −0.179, *p* = 0.002–0.031), whereas triglyceride estimates remained close to the null and nonsignificant throughout (*g* = −0.068 to −0.012, *p* = 0.141–0.783). Varying the assumed within-study correlation produced stable estimates for glucose (ρ = 0.4: *g* = −0.287; ρ = 0.6: *g* = −0.283; ρ = 0.8: *g* = −0.280; *p* = 0.009–0.010) and insulin (ρ = 0.4: *g* = −0.219; ρ = 0.6: *g* = −0.217; ρ = 0.8: *g* = −0.212; *p* = 0.008–0.010). Triglyceride estimates also remained stable and nonsignificant (ρ = 0.4: *g* = −0.035; ρ = 0.6: *g* = −0.036; ρ = 0.8: *g* = −0.036; *p* = 0.464–0.494). These analyses support the robustness of the glucose and insulin findings and the consistently null triglyceride result (Figure 8).

## 4. Discussion

This review suggests that sitting interruption is associated with small acute reductions in glucose and insulin responses, but not with reliable changes in triglyceride-related outcomes, in apparently healthy participants. This cautious interpretation separates a limited short-term glycemic signal from broader glucolipid or long-term cardiometabolic claims that are not supported by the current evidence. A single day of interrupted sitting may modestly attenuate postprandial glucose and insulin responses, but it should not be presented as evidence that all glucolipid outcomes or long-term cardiometabolic risks improve. This distinction is the central contribution of the synthesis.

From a metabolite-oriented perspective, the present findings should be interpreted as changes in a limited set of circulating postprandial metabolic biomarkers, rather than as evidence of a comprehensive shift in the postprandial metabolome. The included outcomes—glucose, insulin, triglycerides, NEFA and C-peptide—represent interrelated but temporally distinct components of substrate handling. Glucose and insulin primarily reflect early postprandial glycemic appearance, skeletal-muscle glucose disposal and pancreatic insulin demand, whereas triglycerides and NEFA reflect slower lipid transport, hydrolysis, fatty-acid uptake, adipose-tissue lipolysis and re-esterification. C-peptide provides complementary information on endogenous insulin secretion but may be less sensitive to brief behavioral perturbations in metabolically healthy participants with preserved β-cell function. This framework helps explain why the strongest signal in the present synthesis was observed for glucose, a smaller signal was observed for insulin, and lipid-related or β-cell secretory markers remained inconclusive. As detailed in the Results, the sparse evidence for NEFA and C-peptide precluded reliable identification of their hierarchical variance structures. Although all effect estimates and uncertainty statistics are reported transparently, the point estimates should be interpreted descriptively, and the associated confidence intervals and *p* values are too unstable to support confirmatory or clinical interpretation.

Clinical relevance should be considered separately for each metabolic outcome. For glucose, a pooled reduction of roughly one-quarter of a standard deviation reflects a small attenuation of the postprandial excursion that, in apparently healthy participants, is unlikely on its own to alter glycemic classification or established cardiometabolic risk stratification; its potential clinical value lies only in the hypothesis that such small acute signals, if repeated and sustained, might accumulate over time, which the present acute evidence cannot establish. The insulin finding is directionally consistent with this interpretation, as a lower insulin exposure alongside a smaller glucose excursion suggests marginally more efficient short-term postprandial handling; however, this remains a subclinical, mechanistic signal rather than evidence of improved insulin sensitivity or reduced insulin resistance in clinical terms. For triglycerides, the absence of a significant pooled effect indicates that sitting interruption should not be regarded as a means of improving acute postprandial lipid metabolism in this population. Taken together, the clinically relevant interpretation is narrow and tentative: a single interrupted-sitting day may produce small improvements in early postprandial glycemic markers, although the evidence is of low certainty; no triglyceride effect was demonstrated, the NEFA and C-peptide findings are hypothesis-generating only, and no inference can be made regarding durable clinical benefit.

The glucose and insulin findings have a plausible mechanistic basis. Skeletal muscle is the main site of postprandial glucose disposal, and contraction activates insulin-dependent and insulin-independent uptake pathways [51]. Even low-intensity movement can recruit glucose transporter type 4 (GLUT4)-related signaling [52]. Prolonged sitting represents a state of markedly reduced skeletal-muscle contractile activity, and even short-term inactivity can impair insulin action [53]. Repeated brief activity breaks may partially reverse this state by recruiting otherwise inactive lower-limb muscle during the absorptive period. Mechanistically, muscle contractions can stimulate glucose uptake through insulin-dependent and insulin-independent pathways, including GLUT4-related signaling and contraction-sensitive pathways such as AMP-activated protein kinase AMPK [54]. This mechanistic pattern is consistent with the small glucose and insulin reductions observed in the present synthesis. However, the pooled effects were small and the certainty of evidence was low; therefore, this pattern should be interpreted as a modest acute metabolic signal rather than as evidence of clinically meaningful glycemic improvement. None of the included trials measured muscle-level signaling, substrate flux or enzyme activity directly; this mechanistic account is therefore inferred from external physiological literature rather than demonstrated by the trials synthesized here. Thus, glucose and insulin provide useful but partial markers of early postprandial glycemic handling; they do not capture the wider metabolic adaptations that may occur across lipid, amino acid or mitochondrial substrate pathways.

The modality pattern is broadly consistent with this interpretation, although the present evidence base cannot formally distinguish between interruption modalities. Standing changes posture, but walking recruits larger lower-limb muscle groups in a more rhythmic pattern. Walking may also support local hemodynamics through the skeletal-muscle pump [55]. This may explain why ambulatory interruptions have shown clearer glucose-related signals than uninterrupted sitting and, where directly compared, than standing-only strategies [33,56]. Although walking had the least sparse evidence in the present synthesis, the subgroup analyses could not establish any modality or intensity as superior. In brief repeated-break protocols, the timing of muscle recruitment relative to meal ingestion, the frequency of contractions, the amount of active muscle mass and real-world feasibility may be as important as nominal exercise intensity when the outcome is short-term postprandial substrate handling rather than exercise performance.

The null or inconsistent lipid-related findings probably reflect different biology and timing rather than a simple absence of metabolic relevance. Postprandial triglycerides depend on delayed production and clearance of triglyceride-rich lipoproteins, including lipoprotein-lipase-regulated pathways [57,58,59]. Unlike contraction-stimulated glucose uptake, which can be activated acutely through GLUT4 translocation, skeletal-muscle lipoprotein lipase activity typically rises only several hours after sustained muscular contraction, so the brief, low-intensity bouts used in sitting-interruption protocols may be insufficient to accelerate triglyceride clearance. Human kinetic data also show that feeding changes apolipoprotein B (ApoB) secretion and catabolism [60]. These processes unfold over hours, so short protocols may alter glucose flux before they measurably change lipid clearance. Evidence from postprandial hypertriglyceridemia similarly suggests that lipid lowering may require a larger or differently timed stimulus [61]. More specifically, postprandial triglyceride concentrations depend on intestinal and hepatic triglyceride-rich lipoprotein appearance, lipoprotein-lipase-mediated hydrolysis, tissue fatty-acid uptake and remnant clearance [62,63,64,65]. These processes may require a larger energy deficit, a delayed measurement window, prior-day activity exposure or different meal-fat conditions than those needed to detect changes in glucose flux [62,63]. NEFA responses are additionally shaped by adipose-tissue lipolysis, insulin-mediated suppression of fatty-acid release and re-esterification, which may fluctuate across the postprandial period. Thus, short same-day protocols in healthy participants may be sufficiently sensitive to detect glucose-insulin changes but insufficient to capture delayed or tissue-specific effects on lipid turnover. This temporal dissociation is also consistent with the Network Physiology of Exercise framework, which conceptualizes responses to exercise as emerging from dynamically coupled physiological subsystems operating across different spatiotemporal scales [66]. From this perspective, repeated activity breaks represent a shared brief physiological perturbation across metabolic systems. The glucose and insulin axis may respond relatively rapidly through glucose disposal mediated by muscle contraction, whereas lipid transport and clearance may require a longer response period and therefore remain unchanged within the acute experimental window. This interpretation is conceptual rather than directly tested in the present review because the included trials did not quantify dynamic coupling among physiological systems.

The subgroup findings do not resolve the prescription question. No moderator family—participant age, interruption modality, exercise intensity or experimental context—significantly modified the pooled response for any outcome once effect-size dependency was modeled with cluster-robust variance estimation at the cohort level. Several categories were represented by only one or two independent cohorts, and their confidence intervals were correspondingly wide; for two moderator families the between-subgroup model did not converge at all. The present evidence base is therefore not informative about which interruption features matter but rather indicates that they do not matter. Because moderators were assessed at the study level, they should guide future trial design rather than clinical rules. Direct within-participant comparisons remain necessary before intensity, modality or frequency can be ranked with confidence. In particular, any directional patterns within subgroups should be interpreted as hypotheses about metabolic responsiveness, not as definitive evidence that one interruption prescription is superior.

Relative to previous reviews, the main advance is not simply an updated pooled estimate. This analysis restricts inference to apparently healthy participants, retains dependent effect sizes through a three-level model, and evaluates glycemic and lipid endpoints together. By preserving repeated measures rather than collapsing them, the model supports a cleaner estimate of acute response while keeping the biological contrast visible. That structure exposes the central contrast: sitting interruption has more consistent acute effects on glucose-insulin handling than on triglyceride metabolism [8,9,14]. Thus, the advance of the present study lies in refining the metabolic and methodological interpretation of sitting-interruption evidence, not in establishing a new broad cardiometabolic intervention claim. The three-level framework is especially relevant for acute metabolism studies because repeated postprandial sampling, multiple outcome definitions and multiple intervention contrasts are common sources of dependent effect sizes. Preserving this structure allows the synthesis to remain closer to the biological time course of postprandial metabolism.

From a practical perspective, and recognizing that the supporting evidence is of low certainty, repeated light ambulatory or muscle-recruiting breaks may be considered when feasible as a low-burden way to reduce uninterrupted sitting time, particularly around meals. Any such suggestion is tentative and should not be presented to patients or the public as an established recommendation. However, these findings should not be interpreted as evidence that sitting interruption replaces structured exercise or confers broad long-term cardiometabolic protection. This aligns with intervention evidence showing that sedentary time can be displaced [15,16,17]. Free-living behavioral interventions further indicate that reducing and interrupting sitting is achievable and can be sustained over weeks to months [15,16,17]. These longer-term studies, however, have generally measured behavioral adherence or intermediate risk markers rather than the acute postprandial responses examined here, so the two studies are best regarded as complementary. The present synthesis characterizes the immediate metabolic signal that a single interrupted-sitting day can produce, whereas free-living trials address whether such behavior can be maintained and whether it translates into durable cardiometabolic benefit. Whether the acute postprandial responses observed here ultimately connect to long-term, free-living health outcomes remains an open question that neither body of evidence can resolve on its own. Sitting interruption is therefore better framed as a low-burden adjunct that targets how inactivity is accumulated across the day, while structured exercise remains necessary for broader fitness and cardiometabolic benefit.

Several limitations temper this interpretation. Certainty was low for the primary outcomes because risk-of-bias concerns and residual inconsistency remain. Participant blinding is intrinsically difficult in behavioral crossover trials because participants necessarily know whether they are sitting continuously or interrupting sitting with activity; this limitation is inherent to the intervention rather than a deficiency of individual studies, and it accounts for most of the ‘some concerns’ judgments in the deviations-from-intended-interventions domain. Its practical impact on the present estimates is, however, likely to be limited: all outcomes analyzed here are objective, laboratory-measured biochemical markers—glucose, insulin, triglycerides, NEFA and C-peptide—rather than self-reported or performance-dependent endpoints, so they are largely insensitive to participants’ awareness of their allocation. The more relevant safeguard in this context is blinding of outcome assessment, which is generally supported by automated, code-labeled biochemical assays. Reporting of randomization procedures and selective-reporting safeguards was nonetheless incomplete in several studies, which contributed to the overall certainty being rated as low. Although most independent cohorts used inter-condition intervals of at least 72 h, residual carryover cannot be excluded completely because two cohorts permitted intervals as short as 24 h and one study did not report the exact inter-visit duration. The marked imbalance across these categories also precluded a meaningful moderator analysis of washout adequacy.

The evidence base is heavily weighted toward young, normal-weight adults: only one included cohort enrolled older adults, one enrolled middle-aged adults, and none included individuals with obesity. This gap is particularly consequential because the populations most likely to benefit clinically are precisely those least represented in the current evidence. We therefore emphasize explicitly that the present findings should not be generalized to populations with obesity, type 2 diabetes or prediabetes, or older age. In these groups, altered baseline insulin sensitivity, β-cell reserve, skeletal-muscle mass, and visceral adiposity may change both the direction and the magnitude of the metabolic response to sitting interruption, and the small effects observed here in young, healthy adults cannot be assumed to apply. Any extension of these conclusions to higher-risk or clinical populations therefore requires dedicated trials conducted specifically in those groups, and, conversely, findings from clinical-population trials should not be extrapolated directly to healthy adults [67]. Inclusion was also restricted to English-language publications. This may have introduced language bias because eligible trials published in other languages were not captured. This is relevant because much of the research in this field is conducted in non-English-speaking regions, and their omission could affect the comprehensiveness and generalizability of the synthesis. Similarly, because all databases were searched with free-text terms only, without database-specific controlled vocabulary (MeSH/Emtree), studies indexed primarily under subject headings but lacking our keywords in the title, abstract, or keyword fields could have been missed; a deliberately broad keyword set and extensive citation searching were used to mitigate—but cannot exclude—this possibility. The estimates should be interpreted as acute controlled-protocol effects, not durable behavioral-intervention effects. Critically, the acute postprandial responses observed here cannot be assumed to predict long-term cardiometabolic adaptations: a single-session metabolic signal does not establish that repeated sitting interruption will produce sustained changes in insulin sensitivity, lipid handling or cardiovascular risk over weeks to years, and this extrapolation would require prospective trials with chronic exposure and hard or intermediate clinical endpoints. The included trials were conducted under a mixture of controlled Laboratory and Simulated real-world conditions; although both were retained to reflect the available evidence, these settings differ in dietary control, activity monitoring and environmental standardization, so effects observed under tightly controlled conditions may not transfer directly to less-controlled, Simulated real-world settings.

Subgroup and meta-regression analyses remain vulnerable to ecological confounding from break timing, habitual activity and fitness. Meal composition was a further source of variability: the type, size, macronutrient balance and timing of the test meals were not standardized across studies, and because postprandial glucose and lipid responses are sensitive to these factors, this between-study variability may have contributed to residual heterogeneity and limited the comparability of pooled estimates. These analyses are further constrained by the small number of independent cohorts contributing to several subgroups and should therefore be interpreted as exploratory rather than confirmatory. In addition, the assessment of small-study effects was constrained by the number of available cohorts: Egger’s regression is underpowered with so few clusters, so the absence of detectable funnel-plot asymmetry provides only weak reassurance against publication or small-study bias.

The present findings should also be distinguished from adjacent but conceptually different evidence streams, including acute cognitive responses to sitting interruptions [68], exercise-related adipokine and inflammatory responses in adults with type 2 diabetes [69], and caffeine-modulated cardiometabolic responses to exercise [70], because these studies concern different populations, exposures, and outcome domains. A further limitation is that the included trials generally reported conventional circulating metabolic biomarkers rather than detailed metabolomic or lipidomic profiles. Therefore, the present synthesis cannot determine whether sitting interruption alters specific lipid species, amino acid metabolites, acylcarnitines or other metabolite signatures that may provide more mechanistic resolution. This limitation is particularly important because circulating glucose, insulin, triglycerides, NEFA and C-peptide represent only a narrow window into the broader postprandial metabolic network.

Future trials should manipulate break intensity, duration, frequency and modality within participants; standardize meals; measure fitness and habitual activity; and extend monitoring to capture lipemic trajectories. Beyond these acute mechanistic questions, longer-term randomized controlled trials are needed to determine whether repeated sitting interruption, sustained over weeks to months, produces durable changes in insulin sensitivity, lipid handling and established cardiometabolic risk markers; such trials would also allow standardized interruption protocols to be tested for adherence and effectiveness under free-living conditions. Protocols should distinguish total sitting time, uninterrupted bout length, total break dose and timing relative to meals. Future studies would better align this field with metabolite-focused science by combining standardized meal challenges, prolonged postprandial sampling, targeted metabolomics, lipidomics, insulin/C-peptide kinetics and markers of skeletal muscle and adipose-tissue substrate handling. Such designs would help determine whether the small glucose-insulin effects observed here correspond to broader shifts in postprandial substrate partitioning or remain limited to conventional glycemic biomarkers.

## 5. Conclusions

This three-level meta-analysis suggests that interrupting prolonged sitting is associated with small acute reductions in glucose and insulin responses among apparently healthy participants. However, the certainty of evidence for these outcomes is low, and the observed effects should be interpreted as modest short-term metabolic signals rather than as evidence of clinically meaningful or durable cardiometabolic benefit. Evidence for triglycerides, NEFA and C-peptide remains inconclusive, with very limited certainty for the exploratory outcomes. Accordingly, sitting interruptions may be considered a low-burden adjunctive behavior for reducing uninterrupted sitting time. However, given the low certainty of the current evidence, the present findings should be interpreted as primarily supporting the hypothesis that interrupting prolonged sitting is a promising behavioral strategy, rather than as an evidence-based clinical recommendation. In other words, the available data are best regarded as hypothesis-generating and directional, and current evidence is insufficient to define an optimal prescription, to establish clinically meaningful benefit, or to support broad claims regarding glucolipid or long-term cardiometabolic improvement. Confirming this hypothesis will require adequately powered trials before any formal clinical or public health recommendation can be made. Future adequately powered crossover trials should directly compare interruption dose, timing, modality and intensity under standardized meal and sampling conditions.

## Figures and Tables

**Table 1:** Summary of included publications, independent cohorts, and sitting-interruption protocol characteristics.

Study	Country	n	Female	Age	BMI	Setting	Break Modality	Intensity	Break Schedule	Experiment Duration (h)
Miyashita et al., 2008 [29]	UK	15	0%	23.4 ± 0.8	23.4 ± 0.6	Laboratory	Walking	Moderate	every 30 min; 10 × 3 min; total 30 min	7
Yap et al., 2015 [30]	Singapore	25	52%	23.0 ± 3.0	21.0 ± 1.7	Laboratory	Walking	High	every 20 min; 3 × 10 min; total 30 min	2.5
Miyashita et al., 2009 [31]	UK	10	0%	24.4 ± 1.4	22.8 ± 0.6	Laboratory	Running	High	every 85 min; 6 × 5 min; total 30 min	9
Kuo et al., 2024 [32]	China	24	46%	31 ± 8	22.7 ± 2.3	Simulated setting	Walking	Low	every 20 min; 15 × 2 min; total 30 min	5.33
Peddie et al., 2013 [33]	New Zealand	70	60%	25.96 ± 5.3	23.66 ± 4.0	Simulated setting	Walking	Low	every 30 min; 18 × 1.67 min; total 30 min	9
Gale et al., 2023 [34]	New Zealand	30	73%	25.4 ± 5.4	29.1 ± 6.6	Simulated setting	Resistance activity	Low	every 30 min; 8 × 3 min; total 24 min	4
Hansen et al., 2016 [35]	Denmark	14	57%	22	23	Laboratory	Walking	Low	every 20 min; 7 × 2 min; total 14 min	2.5
Engeroff et al., 2017 [26] ^b^	Germany	18	100%	25.6 ± 2.6	21.5 ± 2.0	Laboratory	Cycling	High	every 40 min; 5 × 6 min; total 30 min	4
Chueh et al., 2025 [36]	China	18	0%	25 ± 4	23.5 ± 3.2	Laboratory	Walking	Low	every 30 min; 7 × 3 min; total 21 min	3.5
Colvin et al., 2024 [37]	United Kingdom	15	53%	26.00 ± 2.50	24.18 ± 3.24	Laboratory	Yoga	Low	every 30 min; 14 × 3 min; total 42 min	7.5
Ma et al., 2021 [25] ^a^	China	16	56%	24 (21–30)	22.2 ± 2.3	Laboratory	Walking	Moderate	every 30 min; 12 × 3 min; total 36 min	9
Miyashita et al., 2006 [38]	United Kingdom	10	0%	25.0 ± 1.3	25.4 ± 1.2	Laboratory	Running	High	every 30 min; 10 × 3 min; total 30 min	7
McCarthy et al., 2017 [39]	United Kingdom	34	53%	40 ± 9	24.5 ± 3	Laboratory	Walking	Low	every 30 min; 12 × 5 min; total 60 min	7.5
Ak et al., 2025 [40]	Türkiye	12	58%	22 ± 3	23.0 ± 3.6	Simulated real-world setting	Walking	Low	every 30 min; 12 × 2 min; total 24 min	7
Benatti et al., 2017 [41]	Brazil	14	0%	30.1 ± 8.8	24.9 ± 4.3	Laboratory	Standing	Low	every 30 min; 18 × 15 min; total 270 min	9
Miyashita et al., 2016 [42]	Japan	15	100%	68.8 ± 3.2	24.0 ± 2.9	Laboratory	Walking	Low	every 15 min; 20 × 1.5 min; total 30 min	8
Ma et al., 2020 [24] ^a^	China	16	56%	24 (21–30)	22.2 ± 2.3	Laboratory	Walking	Moderate	every 30 min; 12 × 3 min; total 36 min	9
Sperlich et al., 2018 [43]	Germany	12	58%	22 ± 2	21.7 ± 2.1	Simulated real-world setting	Resistance activity	High	every 60 min; 1 × 6 min; total 6 min	3
Champion et al., 2018 [44]	UK	24	50%	Females: 39.5 ± 10.3; Males: 32.0 ± 10.5	Females: 24.8 ± 5.1; Males: 26.6 ± 4.5	Simulated real-world setting	Walking	Low	every 60 min; 6 × 20 min; total 120 min	6.5
Thirunavukkarasu et al., 2025 [45]	India	28	54%	24.1 ± 2.4	22.8 ± 2.4	Simulated real-world setting	Stair climbing	Moderate	every 30 min; 3 × 2 min; total 6 min	2
Altenburg et al., 2019 [46]	The Netherlands	20	0%	19.2 ± 0.6	22.2 ± 2.5	Laboratory	Standing	Low	every 60 min; 4 × 10 min; total 40 min	5
Cho et al., 2020 [47]	South Korea	12	42%	23.5 ± 2.9	23.4 ± 2.7	Laboratory	Stair climbing	High	every 60 min; 4 × 5 min; total 20 min	4
Engeroff et al., 2022 [27] ^b^	Germany	18	100%	25.6 ± 2.6	21.5 ± 2.0	Laboratory	Cycling	High	every 40 min; 5 × 6 min; total 30 min	4
Altenburg et al., 2013 [48]	The Netherlands	11	55%	21.4 ± 0.9	23.2 ± 1.5	Laboratory	Cycling	Moderate	every 60 min; 5 × 8 min; total 40 min	8
Peddie et al., 2021 [49]	New Zealand	18	39%	23.5 ± 5.0	23.7 ± 2.6	Laboratory	Walking	Moderate	every 30 min; 12 × 2 min; total 24 min	6

Note: The 25 included publications represented 23 independent cohorts. ^a^ Ma et al. [24] (2020) and Ma et al. [25] (2021) were companion reports derived from the same randomized crossover cohort. ^b^ Engeroff et al. [26] (2017) and Engeroff et al. [27] (2022) were companion reports derived from the same randomized crossover cohort (ethics approval no. 121/13). Non-duplicated outcome data from these companion publications were retained, but each publication pair was assigned a common cohort identifier and counted as one independent cohort in all meta-analytic models and cohort-level cluster-robust analyses. For the setting moderator, “Simulated” combines the “Simulated setting” and “Simulated real-world setting” descriptors reported in Table 1. n, sample size; BMI, body mass index.

**Table 2:** Summary of pooled acute metabolic effects and certainty of evidence. Values are Hedges’ *g* with 95% confidence intervals and prediction intervals. Negative values favor sitting interruption. NEFA and C-peptide estimates are descriptive only; with three and two independent cohorts, respectively, hierarchical variance was not reliably estimable, and inferential statistics are presented for transparency rather than confirmatory inference. GRADE, Grading of Recommendations Assessment, Development and Evaluation.

Outcome	Hedges’ *g*	95% CI	Prediction Interval	I^2^ L2/L3	*p* Value	GRADE Certainty
Glucose	−0.28	−0.49 to −0.08	−1.15 to 0.59	22.49%/35.56%	0.010	Low
Insulin	−0.22	−0.36 to −0.07	−0.51 to 0.07	10.37%/0%	0.009	Low
Triglycerides	−0.04	−0.14 to 0.07	−0.28 to 0.21	6.33%/0%	0.464	Low
C-peptide	0.40	−6.44 to 7.25	−1.40 to 2.21	7.77%/69.85%	0.591	Very low
NEFA	0.18	−0.19 to 0.56	−0.84 to 1.20	61.33%/0%	0.148	Very low

## Data Availability

The datasets were obtained by contacting Zhengqi Qiu (caspiansev@163.com) and confirming that they will only be used for academic research purposes.

## Supplementary Materials

The following supporting information can be downloaded at: https://www.mdpi.com/article/10.3390/metabo16080528/s1, Figure S1: Risk-of-bias profile showing RoB 2 domain-level and overall judgments across included randomized crossover trials; Table S1: PRISMA 2020 checklist; Table S2: Complete search strategies for electronic databases; Table S3: Characteristics of included studies and sitting-interruption protocols; Table S4: Domain-level and overall RoB 2 judgments; Table S5: Detailed subgroup analyses for glucose, insulin and triglycerides; Table S6: GRADE evidence profile for primary and exploratory outcomes; Table S7: Detailed meta-regression results for glucose, insulin and triglycerides; Table S8: Washout intervals and carryover assessment across included crossover publications.
