# Supplementary material for: Acute Effects of Interrupting Prolonged Sitting on Glucose, Insulin and Lipid Metabolism in Healthy Populations: A Three-Level Meta-Analysis
Source: Metabolites. 2026 Jul 27;16(8):528. doi: 10.3390/metabo16080528 (PMC13515831; doi:10.3390/metabo16080528)
Supplement: Supplementary file 1 [file metabolites-16-00528-s001.zip › metabolites-4438746-supplementary.pdf]

Supplementary Materials

Supplementary Figure S1. Risk-of-bias profile showing RoB 2 domain-level and overall judgments across included randomized crossover trials.

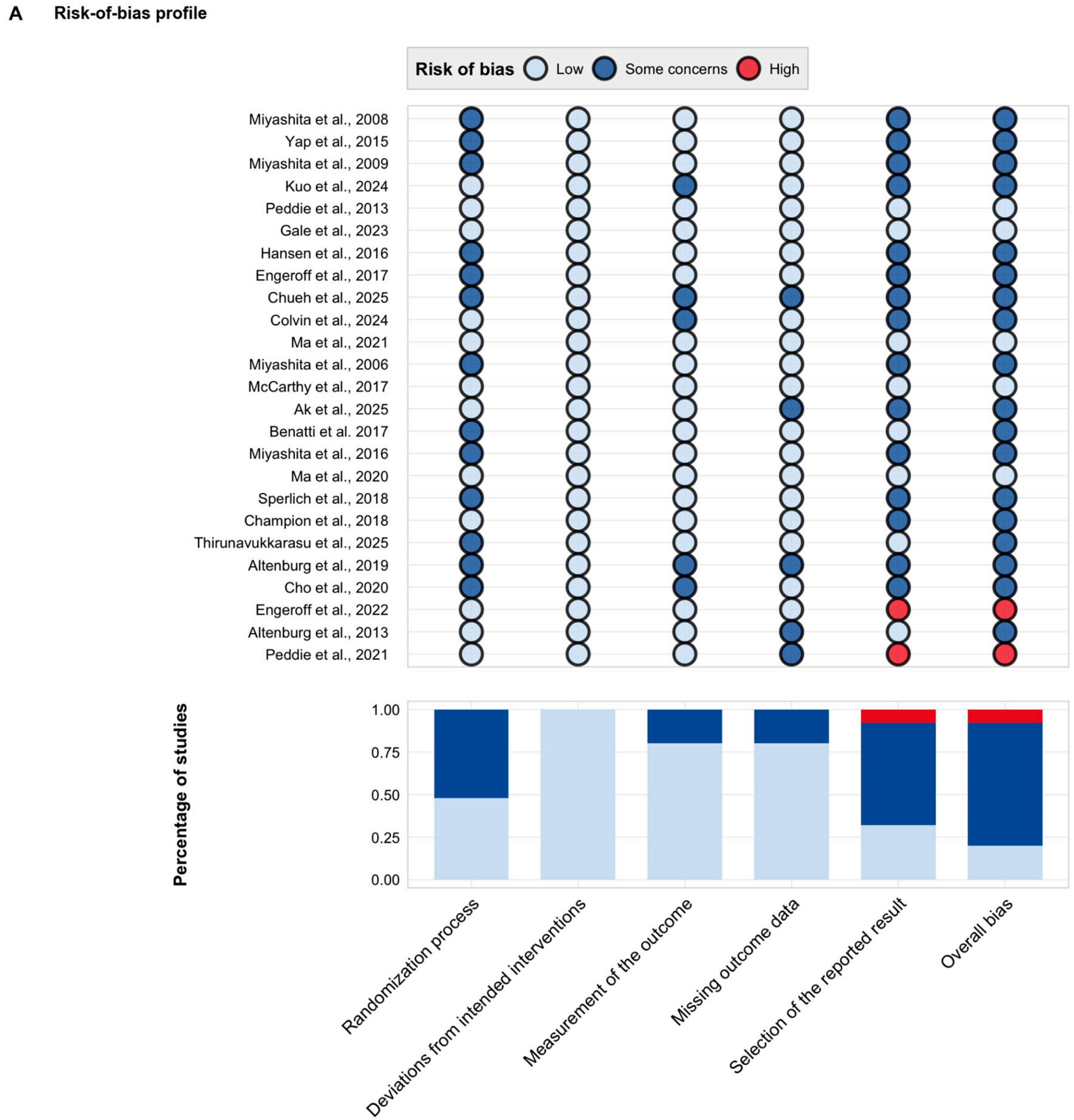

Supplementary Tables

Supplementary Table S1. PRISMA 2020 checklist

Section/topic	Item	Checklist item	Location in manuscript
Title	1	Identify the report as a systematic review.	Title
Abstract	2	Provide a structured summary including background, objectives, eligibility criteria, information sources, risk of bias, synthesis methods, results, limitations, interpretation, registration and funding where applicable.	Abstract
Introduction: Rationale	3	Describe the rationale for the review in the context of existing knowledge.	Introduction
Introduction: Objectives	4	Provide an explicit statement of the objective or question addressed.	Introduction, final paragraph
Methods: Eligibility criteria	5	Specify inclusion and exclusion criteria and how studies were grouped for synthesis.	Methods 2.3
Methods: Information sources	6	Specify databases, registers, websites, organizations, reference lists and other sources searched, and the date each source was last searched.	Methods 2.1
Methods: Search strategy	7	Present the full search strategies for all databases, registers and websites, including filters and limits.	Methods 2.2; Supplementary Table 2
Methods: Selection process	8	Specify methods used to decide whether each record met inclusion criteria, including how many reviewers screened each record and report.	Methods 2.4
Methods: Data collection process	9	Specify methods used to collect data from reports, including how many reviewers collected data and whether investigators were contacted.	Methods 2.5
Methods: Data items	10a	List and define outcomes for which data were sought.	Methods 2.5; Methods 2.7.1
Methods: Data items	10b	List and define other variables for which data were sought and assumptions made.	Methods 2.5; Supplementary Table 3
Methods: Risk of bias	11	Specify methods used to assess risk of bias in included studies.	Methods 2.6; Supplementary Table 4
Methods: Effect measures	12	Specify the effect measure used for each outcome.	Methods 2.7.1
Methods: Synthesis methods	13a-f	Describe processes for deciding eligibility for synthesis, preparing data, tabulating results, choosing synthesis models, exploring heterogeneity and sensitivity analyses.	Methods 2.7.1-2.7.3
Methods: Reporting bias	14	Describe methods used to assess risk of bias due to missing results in a synthesis.	Methods 2.7.3
Methods: Certainty assessment	15	Describe methods used to assess certainty in the body of evidence.	Methods 2.8
Results: Study selection	16a-b	Describe results of the search and selection process, ideally using a flow diagram.	Results 3.1; Figure 1
Results: Study characteristics	17	Cite each included study and present its characteristics.	Results 3.1; Table 1; Supplementary Table 3
Results: Risk of bias	18	Present assessments of risk of bias for each included study.	Results 3.1; Supplementary Figure 2; Supplementary Table 4
Results: Individual studies	19	Present results for each outcome for each study where appropriate.	Figures 1-7; Supplementary Tables 3 and 5
Results: Syntheses	20a-d	Present results of each statistical synthesis, heterogeneity, sensitivity analyses and exploration of causes of heterogeneity.	Results 3.2-3.5; Figures 1-8; Supplementary Table 5
Results: Reporting bias	21	Present assessments of risk of bias due to missing results.	Results 3.5; Figure 7
Results: Certainty of evidence	22	Present assessments of certainty for each outcome.	Results 3.5; Table 2; Supplementary Table 6
Discussion	23a-d	Interpret results, discuss limitations of evidence and review process, and implications for practice, policy and future research.	Discussion
Other information: Registration	24a-c	Provide registration information, protocol availability and amendments.	Methods opening paragraph
Other information: Support	25	Describe sources of financial or non-financial support and funder role.	Funding
Other information: Competing interests	26	Declare competing interests of review authors.	Conflicts of Interest
Other information: Availability of data	27	Report availability of data, code and other materials.	Data Availability Statement; Supplementary Materials

Supplementary Table S2. Complete Search Strategies for Electronic Databases

Overview of database searches

Database	Fields searched	Coverage	Limits / filters	Records identified
PubMed	NCBI; Title/Abstract	Database inception to March 2026	No database date, sex, age, population, or sample-size filters; English full-text peer-reviewed articles retained during screening	3,728
Web of Science Core Collection	Topic search (TS = title, abstract, author keywords, Keywords Plus)	Database inception to March 2026	No database date, sex, age, population, or sample-size filters; English full-text peer-reviewed articles retained during screening	5,276
Cochrane Library	Title, abstract, keyword (:ti,ab,kw)	Database inception to March 2026	No database date, sex, age, population, or sample-size filters; English full-text peer-reviewed articles retained during screening	1,099
Scopus	TITLE-ABS-KEY	Database inception to March 2026	No database date, sex, age, population, or sample-size filters; English full-text peer-reviewed articles retained during screening	6,428
Embase	Elsevier Embase; title/abstract (:ti,ab)	Database inception to March 2026	No database date, sex, age, population, or sample-size filters; English full-text peer-reviewed articles retained during screening	6,533

PubMed (NCBI)

Field	Search details
Database	PubMed (NCBI)
Coverage	Database inception to March 2026
Language/screening limits	No date, sex, age, population, or sample-size limits were applied in the search string. Full-text peer-reviewed journal articles published in English were retained during screening.
Concept 1: sitting/sedentary exposure	"sedentary behavior"[Title/Abstract] OR "sedentary behaviour"[Title/Abstract] OR "sedentary time"[Title/Abstract] OR sitting[Title/Abstract] OR "prolonged sitting"[Title/Abstract] OR "prolonged sedentary"[Title/Abstract] OR "sitting interruption"[Title/Abstract] OR "sedentary interruption"[Title/Abstract] OR break*[Title/Abstract]
Concept 2: metabolic outcomes	glucose[Title/Abstract] OR glycemia[Title/Abstract] OR glycaemia[Title/Abstract] OR insulin[Title/Abstract] OR triglyceride*[Title/Abstract] OR triacylglycerol*[Title/Abstract] OR lipid*[Title/Abstract] OR lipemia[Title/Abstract] OR lipaemia[Title/Abstract] OR "non-esterified fatty acid"[Title/Abstract] OR "non-esterified fatty acids"[Title/Abstract] OR NEFA[Title/Abstract] OR "C-peptide"[Title/Abstract] OR cardiometabolic[Title/Abstract]
Final PubMed search string	(("sedentary behavior"[Title/Abstract] OR "sedentary behaviour"[Title/Abstract] OR "sedentary time"[Title/Abstract] OR sitting[Title/Abstract] OR "prolonged sitting"[Title/Abstract] OR "prolonged sedentary"[Title/Abstract] OR "sitting interruption"[Title/Abstract] OR "sedentary interruption"[Title/Abstract] OR break*[Title/Abstract]) AND (glucose[Title/Abstract] OR glycemia[Title/Abstract] OR glycaemia[Title/Abstract] OR insulin[Title/Abstract] OR triglyceride*[Title/Abstract] OR triacylglycerol*[Title/Abstract] OR lipid*[Title/Abstract] OR lipemia[Title/Abstract] OR lipaemia[Title/Abstract] OR "non-esterified fatty acid"[Title/Abstract] OR "non-esterified fatty acids"[Title/Abstract] OR NEFA[Title/Abstract] OR "C-peptide"[Title/Abstract] OR cardiometabolic[Title/Abstract]))
Records identified	n = 3,728

Embase (Elsevier)

Field	Search details
Database	Embase
Coverage	Database inception to March 2026
Language/screening limits	No date, sex, age, population, or sample-size limits were applied in the search string. Full-text peer-reviewed journal articles published in English were retained during screening.
Concept 1: sitting/sedentary exposure	'sedentary behavior':ti,ab OR 'sedentary behaviour':ti,ab OR 'sedentary time':ti,ab OR sitting:ti,ab OR 'prolonged sitting':ti,ab OR 'prolonged sedentary':ti,ab OR 'sitting interruption':ti,ab OR 'sedentary interruption':ti,ab OR break*:ti,ab
Concept 2: metabolic outcomes	glucose:ti,ab OR glycemia:ti,ab OR glycaemia:ti,ab OR insulin:ti,ab OR triglyceride*:ti,ab OR triacylglycerol*:ti,ab OR lipid*:ti,ab OR lipemia:ti,ab OR lipaemia:ti,ab OR 'non-esterified fatty acid':ti,ab OR 'non-esterified fatty acids':ti,ab OR nefa:ti,ab OR 'c-peptide':ti,ab OR cardiometabolic:ti,ab
Final Embase search string	(('sedentary behavior':ti,ab OR 'sedentary behaviour':ti,ab OR 'sedentary time':ti,ab OR sitting:ti,ab OR 'prolonged sitting':ti,ab OR 'prolonged sedentary':ti,ab OR 'sitting interruption':ti,ab OR 'sedentary interruption':ti,ab OR break*:ti,ab) AND (glucose:ti,ab OR glycemia:ti,ab OR glycaemia:ti,ab OR insulin:ti,ab OR triglyceride*:ti,ab OR triacylglycerol*:ti,ab OR lipid*:ti,ab OR lipemia:ti,ab OR lipaemia:ti,ab OR 'non-esterified fatty acid':ti,ab OR 'non-esterified fatty acids':ti,ab OR nefa:ti,ab OR 'c-peptide':ti,ab OR cardiometabolic:ti,ab))
Records identified	n = 6,533

Web of Science Core Collection

Field	Search details
Database	Web of Science Core Collection
Coverage	Database inception to March 2026
Language/screening limits	No date, sex, age, population, or sample-size limits were applied in the search string. Full-text peer-reviewed journal articles published in English were retained during screening.
Concept 1: sitting/sedentary exposure	TS=("sedentary behavior" OR "sedentary behaviour" OR "sedentary time" OR sitting OR "prolonged sitting" OR "prolonged sedentary" OR "sitting interruption" OR "sedentary interruption" OR break*)
Concept 2: metabolic outcomes	TS=(glucose OR glycemia OR glycaemia OR insulin OR triglyceride* OR triacylglycerol* OR lipid* OR lipemia OR lipaemia OR "non-esterified fatty acid" OR "non-esterified fatty acids" OR NEFA OR "C-peptide" OR cardiometabolic)
Final Web of Science search string	(TS=("sedentary behavior" OR "sedentary behaviour" OR "sedentary time" OR sitting OR "prolonged sitting" OR "prolonged sedentary" OR "sitting interruption" OR "sedentary interruption" OR break*) AND TS=(glucose OR glycemia OR glycaemia OR insulin OR triglyceride* OR triacylglycerol* OR lipid* OR lipemia OR lipaemia OR "non-esterified fatty acid" OR "non-esterified fatty acids" OR NEFA OR "C-peptide" OR cardiometabolic))
Records identified	n = 5,276

Scopus

Field	Search details
Database	Scopus
Coverage	Database inception to March 2026
Language/screening limits	No date, sex, age, population, or sample-size limits were applied in the search string. Full-text peer-reviewed journal articles published in English were retained during screening.
Concept 1: sitting/sedentary exposure	TITLE-ABS-KEY("sedentary behavior" OR "sedentary behaviour" OR "sedentary time" OR sitting OR "prolonged sitting" OR "prolonged sedentary" OR "sitting interruption" OR "sedentary interruption" OR break*)
Concept 2: metabolic outcomes	TITLE-ABS-KEY(glucose OR glycemia OR glycaemia OR insulin OR triglyceride* OR triacylglycerol* OR lipid* OR lipemia OR lipaemia OR "non-esterified fatty acid" OR "non-esterified fatty acids" OR NEFA OR "C-peptide" OR cardiometabolic)
Final Scopus search string	(TITLE-ABS-KEY("sedentary behavior" OR "sedentary behaviour" OR "sedentary time" OR sitting OR "prolonged sitting" OR "prolonged sedentary" OR "sitting interruption" OR "sedentary interruption" OR break*) AND TITLE-ABS-KEY(glucose OR glycemia OR glycaemia OR insulin OR triglyceride* OR triacylglycerol* OR lipid* OR lipemia OR lipaemia OR "non-esterified fatty acid" OR "non-esterified fatty acids" OR NEFA OR "C-peptide" OR cardiometabolic))
Records identified	n = 6,428

Cochrane Library

Field	Search details
Database	Cochrane Library
Coverage	Database inception to March 2026
Language/screening limits	No date, sex, age, population, or sample-size limits were applied in the search string. Full-text peer-reviewed journal articles published in English were retained during screening.
Concept 1: sitting/sedentary exposure	#1 ("sedentary behavior" OR "sedentary behaviour" OR "sedentary time" OR sitting OR "prolonged sitting" OR "prolonged sedentary" OR "sitting interruption" OR "sedentary interruption" OR break*):ti,ab,kw
Concept 2: metabolic outcomes	#2 (glucose OR glycemia OR glycaemia OR insulin OR triglyceride* OR triacylglycerol* OR lipid* OR lipemia OR lipaemia OR "non-esterified fatty acid" OR "non-esterified fatty acids" OR NEFA OR "C-peptide" OR cardiometabolic):ti,ab,kw
Final Cochrane Library search string	#1 ("sedentary behavior" OR "sedentary behaviour" OR "sedentary time" OR sitting OR "prolonged sitting" OR "prolonged sedentary" OR "sitting interruption" OR "sedentary interruption" OR break*):ti,ab,kw<br>#2 (glucose OR glycemia OR glycaemia OR insulin OR triglyceride* OR triacylglycerol* OR lipid* OR lipemia OR lipaemia OR "non-esterified fatty acid" OR "non-esterified fatty acids" OR NEFA OR "C-peptide" OR cardiometabolic):ti,ab,kw<br>#3 #1 AND #2
Records identified	n = 1,099

Additional searches and other sources

Field	Search details
Websites	Records identified from websites: n = 20.
Citation searching	Records identified through citation searching: n = 32.
Snowballing method	Backward citation checking of included articles, forward citation tracking in Google Scholar/Web of Science where available, related-articles searches in MEDLINE and Embase, and manual screening of relevant systematic reviews were used to identify additional eligible trials.
Protocol/register checking	PROSPERO and the Cochrane Database of Systematic Reviews were searched to verify whether a relevant registered or published protocol existed.
Population filters	None. Population eligibility was applied during title/abstract and full-text screening.
Total other-source records identified	n = 52.

Supplementary Table S3. Characteristics of included studies and sitting-interruption protocols

Note. Shaded columns (Break frequency, Number of breaks, Duration per break, and Total break duration) denote the break-dosing parameters of the sitting-interruption protocol. Macronutrient percentages are proportions of total energy unless otherwise specified. ACSM, American College of Sports Medicine; CHO, carbohydrate; GL, glycemic load; HRR, heart rate reserve; NA, not available; NR, not reported; OGTT, oral glucose tolerance test; RPE, rating of perceived exertion.

studyID	Author	Country	Sex ratio (male/female)	%female	Sample size (participants)	Age(mean+sd)	BMI(mean+sd)	Setting	Break modality	Specimen type	Intensity	ACSM category	Break frequency (min)	Number of breaks	Duration per break	Total break duration (min)	Same-day experimental duration (h)	Test meal (composition/energy load)
1	Miyashita et al., 2008	UK	15/0	0	15	23.4 ± 0.8	23.4 ± 0.6	Laboratory	Walking	Plasma	NA	Moderate	30	10	3	30	7	NR
NA	NA	NA	15/0	0	15	23.4 ± 0.8	23.4 ± 0.6	Laboratory	Walking	Plasma	NA	Moderate	NA	1	30	30	7	
2	Yap et al., 2015	Singapore	12/13	0.52	25	23.0±3.0	21.0±1.7	Laboratory	Walking	Plasma	70%VO2max	High	20	3	10	30	2.5	75-g OGTT
NA	NA	NA	12/13	0.52	25	23.0±3.0	21.0±1.7	Laboratory	Walking	Plasma	70%VO2max	High	NA	1	30	30	2.5	
3	Miyashita et al., 2009	UK	10/0	0	10	24.4 ± 1.4	22.8 ± 0.6	Laboratory	Running	Mixed	70%VO2max	High	85	6	5	30	9	NR
4	Kuo et al., 2024	China	13/11	0.46	24	31 ± 8	22.7 ± 2.3	Simulated setting	Walking	Whole blood	RPE = 10(Borg 6–20)	Low	20	15	2	30	5.33	Mixed meal, 8.8 kcal/kg (77% CHO, 16% fat, 7% protein)
5	Peddie et al., 2013	New Zealand	28/42	0.6	70	25.96± 5.3	23.66± 4.0	Simulated setting	Walking	Plasma	45% maximal aerobic capacity	Low	30	18	1.67	30	9	Meal-replacement drink: 1.12 g CHO, 0.46 g fat, and 0.54 g protein/kg
NA	NA	NA	NA	NA	NA	NA	NA	Simulated setting	Walking	Plasma	60% maximal aerobic capacity	Moderate	NA	1	30	30	9	
6	Gale et al., 2023	New Zealand	8/22	0.73	30	25.4±5.4	29.1±6.6	Simulated setting	Resistance activity	Plasma	N/A	Low	30	8	3	24	4	Two standardized mixed meals (composition/load NR)
7	Hansen et al., 2016	Denmark	6/8	0.57	14	22	23	Laboratory	Walking	Whole blood	N/A	Low	20	7	2	14	2.5	High-fat drink, ~736 kcal (50 g fat, 69.9 g CHO)
8	Engeroff et al., 2017	Germany	0/18	1	18	25.6 ± 2.6	21.5 ± 2.0	Laboratory	Cycling	Serum	70% VO2max	High	40	5	6	30	4	Standardized breakfast (66 g CHO, 20 g fat, 18 g protein)
NA	NA	NA	0/18	1	18	25.6 ± 2.6	21.5 ± 2.0	Laboratory	Cycling	Serum	70% VO2max	High	NA	1	30	30	4	
9	Chueh et al., 2025	China	18/0	0	18	25 ± 4	23.5 ± 3.2	Laboratory	Walking	Whole blood	6.4 km/h	Low	30	7	3	21	3.5	Standardized meal (composition/load NR)
10	Colvin et al., 2024	United Kingdom	7/8	0.53	15	26.00±2.50	24.18±3.24	Laboratory	Yoga	Whole blood	MET = 2.5	Low	30	14	3	42	7.5	Standardized breakfast and lunch (composition/load NR)
NA	NA	NA	7/8	0.53	15	26.00±2.50	24.18±3.24	Laboratory	Tai Chi	Whole blood	MET = 3.0	Low	30	14	3	42	7.5	
11	Ma et al., 2021	China	7/9	0.56	16	24 (21–30)	22.2±2.3	Laboratory	Walking	Serum	60% VO2max	Moderate	30	12	3	36	9	Individualized Chinese meals (~49% CHO, 37% fat, 14% protein)
NA	NA	NA	NA	NA	NA	NA	NA	Laboratory	Walking	Serum	60% VO2max	Moderate	45	9	5	45	9	
NA	NA	NA	NA	NA	NA	NA	NA	Laboratory	Walking	Serum	60% VO2max	Moderate	60	7	8	56	9	
12	Miyashita et al., 2006	United Kingdom	10/0	0	10	25.0 ± 1.3	25.4 ± 1.2	Laboratory	Running	Plasma	70%VO2max	High	30	10	3	30	7	High-fat breakfast/lunch, 46 kJ/kg (~56% fat, 33% CHO, 11% protein)
NA	NA	NA	NA	NA	NA	NA	NA	Laboratory	Running	Plasma	70%VO2max	High	NA	1	30	30	7	
13	McCarthy et al., 2017	United Kingdom	16/18	0.53	34	40±9	24.5 ± 3	Laboratory	Walking	Plasma	3 km/h	Low	30	12	5	60	7.5	Two identical meals, 8 kcal/kg each (51% CHO, 35% fat, 14% protein)
14	Ak et al., 2025	Türkiye	5/7	0.58	12	22 ± 3	23.0 ± 3.6	Simulated real-world setting	Walking	Mixed	35%–45% HRR	Low	30	12	2	24	7	Standardized breakfast/lunch (55–60% CHO, 25–30% fat, 12–15% protein)
NA	NA	NA	5/7	0.58	12	22 ± 3	23.0 ± 3.6	Simulated real-world setting	Walking	Mixed	35%–45% HRR	Low	60	6	4	24	7	
NA	NA	NA	5/7	0.58	12	22 ± 3	23.0 ± 3.6	Simulated real-world setting	Walking	Mixed	35%–45% HRR	Low	120	3	8	24	7	
15	Benatti et al., 2017	Brazil	14/0	0	14	30.1 ± 8.8	24.9 ± 4.3	Laboratory	Standing	Plasma	N/A	Low	30	18	15	270	9	Three standardized meals (GL 75/54/38; 55% CHO, 30% fat, 15% protein)
NA	NA	NA	NA	NA	NA	NA	NA	Laboratory	Walking	Plasma	50%–55% VO2max	Moderate	NA	1	30	30	9	
NA	NA	NA	NA	NA	NA	NA	NA	Laboratory	Walking	Plasma	50%–55% VO2max	Moderate	30	16	15	240	9	
16	Miyashita et al., 2016	Japan	0/15	1	15	68.8±3.2	24.0 ± 2.9	Laboratory	Walking	Mixed	3.7 ± 1.1 km/h	Low	15	20	1.5	30	8	Standardized breakfast/lunch, 38 kJ/kg (~50% CHO, 35% fat, 15% protein)
NA	NA	NA	NA	NA	NA	NA	NA	Laboratory	Walking	Mixed	3.7 ± 1.1 km/h	Low	NA	1	30	30	8	
17	Ma et al., 2020	China	7/9	0.56	16	24 (21–30)	22.2 ± 2.3	Laboratory	Walking	Serum	60% V̇O2max	Moderate	30	12	3	36	9	Individualized Chinese meals (~49% CHO, 37% fat, 14% protein)
NA	NA	NA	7/9	0.56	16	24 (21–30)	22.2 ± 2.3	Laboratory	Walking	Serum	60% V̇O2max	Moderate	45	9	5	45	9	

studyID	Author	Country	Sex ratio (male/female)	%female	Sample size (participants)	Age(mean+sd)	BMI(mean+sd)	Setting	Break modality	Specimen type	Intensity	ACSM category	Break frequency (min)	Number of breaks	Duration per break	Total break duration (min)	Same-day experimental duration (h)	Test meal (composition/energy load)
NA	NA	NA	7/9	0.56	16	24 (21–30)	22.2 ± 2.3	Laboratory	Walking	Serum	60% V̇O2max	Moderate	60	7	8	56	9	
18	Sperlich et al., 2018	Germany	5/7	0.58	12	22 ± 2	21.7 ± 2.1	Simulated real-world setting	Resistance activity	Whole blood	84 ± 7% peak VO2	High	60	1	6	6	3	Standardized breakfast, 11.0 ± 5.4 kcal/kg (macronutrients NR)
19	Champion et al., 2018	UK	12/12	0.5	24	Females：39.5 ± 10.3; Males：32.0 ± 10.5	Females：24.8 ± 5.1; Males：26.6 ± 4.5	Simulated real-world setting	Walking	Whole blood	1.2–3.5 km/h	Low	60	6	20	120	6.5	Two meals, 30% daily energy each (breakfast 57/29/14%; lunch 47/39/14% CHO/fat/protein)
20	Thirunavukkarasu et al., 2025	India	13/15	0.537	28	24.1 ± 2.4	22.8 ± 2.4	Simulated real-world setting	Stair climbing	Whole blood	RPE 12–13	Moderate	30	3	2	6	2	Indian lunch, 33% daily energy (51% CHO, 31% fat, 18% protein)
21	Altenburg et al., 2019	The Netherlands	20/0	0	20	19.2 ±0.6	22.2 ± 2.5	Laboratory	Standing	Mixed	N/A	Low	60	4	10	40	5	Liquid high-fat meal, 843 kcal (58.8 g fat, 92.0 g CHO, 15.6 g protein)
22	Cho et al., 2020	South Korea	7/5	0.42	12	23.5 ± 2.9	23.4 ± 2.7	Laboratory	Stair climbing	Plasma	66% HRR	High	60	4	5	20	4	High-fat meal, 1332 kcal (71 g fat, 83 g CHO, 45 g protein)
23	Engeroff et al., 2022	Germany	0/18	1	18	25.6 ± 2.6	21.5 ± 2.0	21.5 ± 2.0	Cycling	Mixed	70% VO2max	High	40	5	6	30	4	Standardized breakfast (66 g CHO, 20 g fat, 18 g protein)
NA	NA	NA	0/18	1	18	25.6 ± 2.6	20.5–22.5	Laboratory	Cycling	Mixed	70% VO2max	High	NA	1	30	30	4	
24	Altenburg et al., 2013	The Netherlands	5/6	0.55	11	21.4±0.9	23.2±1.5	Laboratory	Cycling	Whole blood	52.0 ± 3.2% HRR	Moderate	60	5	8	40	8	High-fat breakfast/lunch, 843/1190 kcal (58.8/77.1 g fat; 92.0/116.7 g CHO; 15.6/27.7 g protein)
25	Peddie et al., 2021	New Zealand	11/7	0.39	18	23.5±5.0	23.7±2.6	Laboratory	Walking	Plasma	5 km/h	Moderate	30	12	2	24	6	Breakfast + snack, 4697 kJ (170 g CHO [62% energy], 35 g fat [28% energy])

Supplementary Table S4. Domain-level and overall RoB 2 judgments

Study	Randomization process	Deviations from intended interventions	Measurement of the outcome	Missing outcome data	Selection of the reported result	Overall bias
Miyashita et al., 2008	Some concerns	Low	Low	Low	Some concerns	Some concerns
Yap et al., 2015	Some concerns	Low	Low	Low	Some concerns	Some concerns
Miyashita et al., 2009	Some concerns	Low	Low	Low	Some concerns	Some concerns
Kuo et al., 2024	Low	Low	Some concerns	Low	Some concerns	Some concerns
Peddie et al., 2013	Low	Low	Low	Low	Low	Low
Gale et al., 2023	Low	Low	Low	Low	Low	Low
Hansen et al., 2016	Some concerns	Low	Low	Low	Some concerns	Some concerns
Engeroff et al., 2017	Some concerns	Low	Low	Low	Some concerns	Some concerns
Chueh et al., 2025	Some concerns	Low	Some concerns	Some concerns	Some concerns	Some concerns
Colvin et al., 2024	Low	Low	Some concerns	Low	Some concerns	Some concerns
Ma et al., 2021	Low	Low	Low	Low	Low	Low
Miyashita et al., 2006	Some concerns	Low	Low	Low	Some concerns	Some concerns
McCarthy et al., 2017	Low	Low	Low	Low	Low	Low
Ak et al., 2025	Low	Low	Low	Some concerns	Some concerns	Some concerns
Benatti et al., 2017	Some concerns	Low	Low	Low	Low	Some concerns
Miyashita et al., 2016	Some concerns	Low	Low	Low	Some concerns	Some concerns
Ma et al., 2020	Low	Low	Low	Low	Low	Low
Sperlich et al., 2018	Some concerns	Low	Low	Low	Some concerns	Some concerns
Champion et al., 2018	Low	Low	Low	Low	Some concerns	Some concerns
Thirunavukkarasu et al., 2025	Some concerns	Low	Low	Low	Low	Some concerns
Altenburg et al., 2019	Some concerns	Low	Some concerns	Some concerns	Some concerns	Some concerns
Cho et al., 2020	Some concerns	Low	Some concerns	Low	Some concerns	Some concerns
Engeroff et al., 2022	Low	Low	Low	Low	High	High
Altenburg et al., 2013	Low	Low	Low	Some concerns	Low	Some concerns
Peddie et al., 2021	Low	Low	Low	Some concerns	High	High

Supplementary Table S5. Detailed subgroup analyses for glucose, insulin and triglycerides

Glucose subgroup analyses

Subgroup	*K* (*N*)	*Hedges' g*	95% CI	P_d	Power(%)	P_m
**Age**						0.230
Young adults	124 (20)	-0.23	-0.42 to -0.05	0.016	75.4	
Middle-aged adults	5 (2)	-0.96	-11.37 to 9.45	0.450	21.6	
Older adults	6 (1)	0.04	-0.17 to 0.26	0.247	68.5	
Modality						n/e
Walking	70 (12)	-0.38	-0.70 to -0.07	0.022	76.5	
Standing	26 (2)	-0.07	-2.11 to 1.97	0.747	7.0	
Stair climbing	9 (2)	-0.13	-5.69 to 5.44	0.822	6.0	
Resistance	16 (2)	-0.38	-5.39 to 4.63	0.513	16.0	
Mind-body	6 (1)	-0.71	-16.21 to 14.78	0.663	9.0	
Cycling	3 (2)	0.43	-11.33 to 12.19	0.723	7.5	
Running	5 (2)	-0.23	-2.32 to 1.86	0.396	28.7	
**Setting**						0.633
Simulated	30 (7)	-0.34	-0.61 to -0.07	0.021	87.7	
Laboratory	105 (16)	-0.25	-0.56 to 0.05	0.099	42.1	
**Intensity**						0.162
High	26 (6)	-0.05	-0.32 to 0.22	0.670	7.4	
Moderate	47 (5)	-0.16	-0.92 to 0.60	0.578	9.4	
Low	62 (12)	-0.44	-0.76 to -0.13	0.011	87.2	

Note: ***K*** indicates the number of effect sizes/comparisons, and ***N*** indicates the number of independent cohorts included in each subgroup. Subgroup estimates based on fewer than five independent cohorts are exploratory and hypothesis-generating. ***CI*** = confidence interval; P_d = p value for the pooled effect within each subgroup; P_m = p value for differences between subgroups; n/e = not estimable. Moderator-level n/e indicates that the between-subgroup model could not be estimated under cohort-level cluster-robust variance estimation because of sparse categories; subgroup-level n/e indicates that the corresponding statistic could not be estimated.

Insulin subgroup analyses

Subgroup	*K* (*N*)	*Hedges' g*	95% CI	P_d	Power(%)	P_m
**Age**						0.281
Young adults	42 (9)	-0.25	-0.41 to -0.10	0.007	98.3	
Middle-aged adults	5 (2)	-0.11	-3.42 to 3.21	0.749	7.0	
Older adults	2 (1)	-0.05	-0.05 to -0.05	< 0.001	100.0	
Modality						0.864
Walking	17 (6)	-0.17	-0.38 to 0.04	0.087	62.3	
Standing	22 (2)	-0.09	-0.72 to 0.54	0.324	43.3	
Resistance	2 (1)	-0.55	-0.57 to -0.54	0.001	100.0	
Cycling	3 (1)	-0.57	-1.01 to -0.14	0.038	100.0	
Running	5 (2)	-0.06	-1.67 to 1.56	0.728	7.4	
**Setting**						0.853
Simulated	8 (3)	-0.23	-0.91 to 0.44	0.249	38.9	
Laboratory	41 (9)	-0.20	-0.36 to -0.05	0.016	87.9	
**Intensity**						0.184
High	14 (4)	-0.27	-0.69 to 0.15	0.122	64.7	
Moderate	2 (1)	-0.05	-0.05 to -0.05	< 0.001	100.0	
Low	33 (7)	-0.21	-0.43 to 0.01	0.059	68.9	

Note: ***K*** indicates the number of effect sizes/comparisons, and ***N*** indicates the number of independent cohorts included in each subgroup. Subgroup estimates based on fewer than five independent cohorts are exploratory and hypothesis-generating. ***CI*** = confidence interval; P_d = p value for the pooled effect within each subgroup; P_m = p value for differences between subgroups; n/e = not estimable. Moderator-level n/e indicates that the between-subgroup model could not be estimated under cohort-level cluster-robust variance estimation because of sparse categories; subgroup-level n/e indicates that the corresponding statistic could not be estimated.

Triglyceride subgroup analyses

Subgroup	*K* (*N*)	*Hedges' g*	95% CI	P_d	Power(%)	P_m
**Age**						0.635
Young adults	50 (10)	-0.03	-0.16 to 0.11	0.658	7.5	
Middle-aged adults	4 (1)	-0.06	-0.22 to 0.10	0.131	99.8	
Older adults	6 (1)	-0.12	-0.74 to 0.50	0.249	67.8	
Modality						n/e
Walking	20 (5)	-0.11	-0.29 to 0.07	0.150	49.8	
Standing	22 (2)	0.23	-0.88 to 1.33	0.235	73.1	
Resistance	8 (1)	0.09	-0.38 to 0.56	0.245	69.4	
Cycling	1 (1)	-0.07	not estimable	n/e	100.0	
Running	5 (2)	-0.24	-0.80 to 0.32	0.116	100.0	
Stair climbing	4 (1)	-0.08	-0.43 to 0.26	0.205	85.2	
**Setting**						0.740
Simulated	17 (4)	-0.02	-0.17 to 0.13	0.673	7.7	
Laboratory	43 (8)	-0.05	-0.28 to 0.17	0.577	9.0	
**Intensity**						**n/e**
High	10 (4)	-0.15	-0.30 to 0.01	0.061	84.4	
Moderate	5 (1)	-0.37	-1.30 to 0.56	0.124	99.9	
Low	45 (7)	0.02	-0.11 to 0.15	0.687	7.1	

Note: ***K*** indicates the number of effect sizes/comparisons, and ***N*** indicates the number of independent cohorts included in each subgroup. Subgroup estimates based on fewer than five independent cohorts are exploratory and hypothesis-generating. ***CI*** = confidence interval; P_d = p value for the pooled effect within each subgroup; P_m = p value for differences between subgroups; n/e = not estimable. Moderator-level n/e indicates that the between-subgroup model could not be estimated under cohort-level cluster-robust variance estimation because of sparse categories; subgroup-level n/e indicates that the corresponding statistic could not be estimated.

Supplementary Table S6. GRADE evidence profile for primary and exploratory outcomes

Outcome	Effect sizes (cohorts)	GRADE Evidence Certainty Assessment					Hedges' *g* (95% CI)	Certainty†
		Risk of bias	Inconsistency	Indirectness	Imprecision	Other considerations		
**Glucose**	**135 (23 cohorts)**	**Serious**	**Serious**	**Not serious**	**Not serious**	**None**	**−0.28 (−0.49 to −0.08)**	⊕⊕◯◯ **Low**
**Insulin**	**49 (12 cohorts)**	**Serious**	**Serious**	**Not serious**	**Not serious**	**None**	**−0.22 (−0.36 to −0.07)**	⊕⊕◯◯ **Low**
**Triglycerides**	**60 (12 cohorts)**	**Serious**	**Not serious**	**Not serious**	**Serious**	**None**	**−0.04 (−0.14 to 0.07)**	⊕⊕◯◯ **Low**
**NEFA**	**47 (3 cohorts)**	**Serious**	**Serious**	**Not serious**	**Serious**	**None**	0.18 (−0.19 to 0.56)	⊕◯◯◯ **Very low**
**C-peptide**	**23 (2 cohorts)**	**Serious**	**Serious**	**Not serious**	**Serious**	**None**	0.40 (−6.44 to 7.25)	⊕◯◯◯ **Very low**

† GRADE levels of certainty. High: We are very confident that the true effect lies close to the estimate. **Moderate:** The true effect is likely close to the estimate, but there is a possibility it is substantially different. **Low:** Our confidence in the effect estimate is limited—the true effect may be substantially different. **Very low:** We have very little confidence in the effect estimate.

Abbreviations: CI, confidence interval; NEFA, non-esterified fatty acids; cohort, independent randomized crossover trial (companion publications reporting the same cohort are counted once).

Supplementary Table S7. Detailed meta-regression results for glucose, insulin and triglycerides

Outcome	Moderator	k	N	β (beta)	SE	95% CI	t	df	p
**Glucose**	Age (years)	135	23	-0.0071	0.0247	-0.1381 to 0.1238	-0.29	1.65	0.805
	BMI (kg/m2)	135	23	-0.1115	0.0640	-0.2604 to 0.0373	-1.74	7.60	0.121
	Female (% female)	135	23	-0.0008	0.0024	-0.0063 to 0.0046	-0.35	8.14	0.736
	Frequency (min between breaks)	135	23	0.0012	0.0027	-0.0080 to 0.0104	0.46	2.68	0.683
	Interruptions (number of breaks)	135	23	-0.0099	0.0127	-0.0390 to 0.0192	-0.78	8.36	0.457
	Break duration (min per break)	135	23	0.0057	0.0109	-0.0262 to 0.0376	0.52	3.51	0.632
	Trial duration (h)	135	23	-0.0041	0.0371	-0.0857 to 0.0775	-0.11	11.10	0.913
**Insulin**	Age (years)	49	12	0.0046	0.0038	-0.0125 to 0.0217	1.22	1.90	0.352
	BMI (kg/m2)	49	12	0.0042	0.0530	-0.1443 to 0.1528	0.08	3.90	0.940
	Female (% female)	49	12	-0.0035	0.0015	-0.0070 to 0.0001	-2.32	6.51	0.056
	Frequency (min between breaks)	49	12	0.0052	0.0031	-0.0035 to 0.0138	1.69	3.85	0.169
	Interruptions (number of breaks)	49	12	-0.0003	0.0085	-0.0231 to 0.0225	-0.03	4.34	0.975
	Break duration (min per break)	49	12	0.0191	0.0080	-0.0085 to 0.0467	2.38	2.64	0.109
	Trial duration (h)	49	12	0.0311	0.0277	-0.0475 to 0.1096	1.12	3.80	0.328
**Triglycerides**	Age (years)	60	12	-0.0024	0.0025	-0.0182 to 0.0134	-0.95	1.44	0.473
	BMI (kg/m2)	60	12	0.0002	0.0572	-0.1521 to 0.1524	0.00	4.49	0.998
	Female (% female)	60	12	-0.0000	0.0019	-0.0046 to 0.0045	-0.02	6.64	0.985
	Frequency (min between breaks)	60	12	0.0010	0.0012	-0.0036 to 0.0057	0.85	2.33	0.471
	Interruptions (number of breaks)	60	12	-0.0045	0.0066	-0.0215 to 0.0125	-0.68	4.97	0.528
	Break duration (min per break)	60	12	0.0081	0.0099	-0.0288 to 0.0450	0.82	2.37	0.488
	Trial duration (h)	60	12	-0.0269	0.0218	-0.0879 to 0.0340	-1.24	3.92	0.285

Note: All models are three-level random-effects meta-regressions fitted by restricted maximum likelihood (metafor::rma.mv) with a working variance–covariance matrix assuming a within-study correlation of $\rho = 0.6$; coefficients, standard errors, confidence intervals, test statistics and p values are derived from cluster-robust variance estimation with the CR2 small-sample correction at the cohort level (clubSandwich::coef_test), with Satterthwaite degrees of freedom. Each moderator was mean-centred and fitted in a separate model. k = number of effect sizes; N = number of independent cohorts; β = regression coefficient per one-unit increase in the moderator; CI = confidence interval; df = Satterthwaite degrees of freedom. Negative β values indicate that higher moderator values are associated with more negative (more favourable) effect sizes. No moderator reached statistical significance for any outcome. Because the number of independent cohorts is small (12–23), all meta-regression analyses are exploratory and are reported for completeness and transparency rather than as confirmatory tests.

Supplementary Table S8. Washout intervals and carryover assessment across included crossover publications

Publication	Washout interval	Assessment
Miyashita et al., 2008	≥7 d	Adequate
Yap et al., 2015	3–10 d; women: cycle days 7–14	Adequate
Miyashita et al., 2009	≥7 d	Adequate
Kuo et al., 2024	≥24 h; all conditions within 7 d	**Potential concern**
Peddie et al., 2013	≥6 d; median 6 d (IQR 6–13)	Adequate
Gale et al., 2023	≥6 d; median 6 d (IQR 6–13); order-adjusted	Adequate
Hansen et al., 2016	≥4 d	Adequate
Engeroff et al., 2017[1]	≥7 d; balanced sequence	Adequate
Chueh et al., 2025	≥24 h	**Potential concern**
Colvin et al., 2024	Separate visits; interval NR; overnight fast before each visit	**Unclear**
Ma et al., 2021[2]	5–14 d	Adequate
Miyashita et al., 2006	7 d	Adequate
McCarthy et al., 2017	≥7 d	Adequate
Ak et al., 2025	≥4 d	Adequate
Benatti et al., 2017	5–15 d	Adequate
Miyashita et al., 2016	≥7 d	Adequate
Ma et al., 2020[2]	5–14 d	Adequate
Sperlich et al., 2018	≥72 h	Adequate
Champion et al., 2018	≥6 d	Adequate
Thirunavukkarasu et al., 2025	≥6 d	Adequate
Altenburg et al., 2019	≥6 d	Adequate
Cho et al., 2020	≥72 h	Adequate
Engeroff et al., 2022[1]	7–21 d	Adequate
Altenburg et al., 2013	≥7 d	Adequate
Peddie et al., 2021	≥4 d; median 7 d (IQR 6–20)	Adequate

Note. d, days; h, hours; IQR, interquartile range; NR, not reported. [1]Engeroff et al. (2017/2022) and [2]Ma et al. (2020/2021) were companion publications from the same cohorts. Intervals ≥72 h were considered adequate; 24-h minimum intervals were classified as potential concerns; separate visits with an unreported interval were classified as unclear.
